# Limited Role of Murine ATM in Oncogene-Induced Senescence and p53-Dependent Tumor Suppression

**DOI:** 10.1371/journal.pone.0005475

**Published:** 2009-05-07

**Authors:** Alejo Efeyan, Matilde Murga, Barbara Martinez-Pastor, Ana Ortega-Molina, Rebeca Soria, Manuel Collado, Oscar Fernandez-Capetillo, Manuel Serrano

**Affiliations:** 1 Tumor Suppression Group, Spanish National Cancer Research Centre (CNIO), Madrid, Spain; 2 Genomic Instability Group, Spanish National Cancer Research Centre (CNIO), Madrid, Spain; Ordway Research Institute, United States of America

## Abstract

Recent studies in human fibroblasts have provided a new general paradigm of tumor suppression according to which oncogenic signaling produces DNA damage and this, in turn, results in ATM/p53-dependent cellular senescence. Here, we have tested this model in a variety of murine experimental systems. Overexpression of oncogenic Ras in murine fibroblasts efficiently induced senescence but this occurred in the absence of detectable DNA damage signaling, thus suggesting a fundamental difference between human and murine cells. Moreover, lung adenomas initiated by endogenous levels of oncogenic K-Ras presented abundant senescent cells, but undetectable DNA damage signaling. Accordingly, K-Ras-driven adenomas were also senescent in *Atm*-null mice, and the tumorigenic progression of these lesions was only modestly accelerated by *Atm*-deficiency. Finally, we have examined chemically-induced fibrosarcomas, which possess a persistently activated DNA damage response and are highly sensitive to the activity of p53. We found that the absence of *Atm* favored genomic instability in the resulting tumors, but did not affect the persistent DNA damage response and did not impair p53-dependent tumor suppression. All together, we conclude that oncogene-induced senescence in mice may occur in the absence of a detectable DNA damage response. Regarding murine Atm, our data suggest that it plays a minor role in oncogene-induced senescence or in p53-dependent tumor suppression, being its tumor suppressive activity probably limited to the maintenance of genomic stability.

## Introduction

ATM plays an important role in tumor suppression as indicated by the *in vivo* consequences of ATM deficiency both in human and mice. In particular, genetic deficiency of *ATM* in humans is responsible for the ataxia-telangiectasia syndrome characterized by a high susceptibility to both DNA damage and cancer [1], [2]. Mice genetically deficient in *Atm* present early onset of thymic lymphomas [3]–[5], and enhanced susceptibility to a variety of experimentally induced cancers, such as, mammary tumors, skin tumors, B-cell lymphomas, intestinal tumors, and others [6]–[13].

At a molecular level, ATM is thought to contribute to cancer protection through two main mechanisms. On one hand, ATM acts locally at the sites of double-strand DNA breaks promoting repair [14]; and, on the other hand, ATM activates p53, directly or through its downstream effector kinase CHK2, promoting p53-dependent responses, such as transient cell cycle arrest, senescence or apoptosis [15], [16]. Each of the above-mentioned activities of ATM, namely, DNA repair and p53 activation, could contribute independently to tumor suppression. The activation of p53 by ATM has gained great relevance lately as a general tumor suppressive mechanism after the proposal of a paradigm meant to apply to the majority of cancers [17], [18]. According to this model, oncogenic signaling in emerging tumor cells induces *per se* sufficient DNA damage to trigger p53-dependent oncogene-induced senescence. This model is based on two main sets of evidences. First, a variety of human tumors (including bladder, lung, breast and colon tumors, as well as melanoma) at their earliest detectable stages present molecular markers indicative of an ongoing DNA damage response, including phosphorylated ATM [19], [20]. Second, inactivation of individual components of the DNA damage response, such as ATM, in cultured human fibroblasts eliminates oncogene-induced senescence and renders these cells permissive to oncogene-driven proliferation [21]–[23].

Previous work on murine models has put forward an alternative model to explain oncogene-induced p53 activation, being its main features that it is independent of DNA damage and that it places Arf as the critical sensor of oncogenic signaling that mediates p53 activation [24], [25]. This model is well supported by *in vivo* genetic evidence in mouse cancer models. For example, Arf is essential for the upregulation of p53 in pre-malignant skin tumors [26], for p53-mediated tumor suppression in lymphomas and sarcomas [27], [28], and for oncogene-induced senescence during mammary tumorigenesis [29], [30]. Conceivably, this Arf-based model operating during murine tumorigenesis could coexist with an Atm-based model similar to the one proposed in human tumorigenesis. To test this possibility, we have examined genetically in mice the role of Atm in oncogene-induced senescence and p53-dependent tumor suppression. For this, we have used experimental systems that are well suited to study oncogene-induced senescence and p53-dependent tumor suppression. In this regard, it is important to clarify that our experiments are not designed to address the well-established role of Atm in tumor suppression through the maintenance of genomic stability by DNA repair.

## Results

### Oncogenic signaling by Ras does not activate Atm in mouse fibroblasts

**Figure 1:**
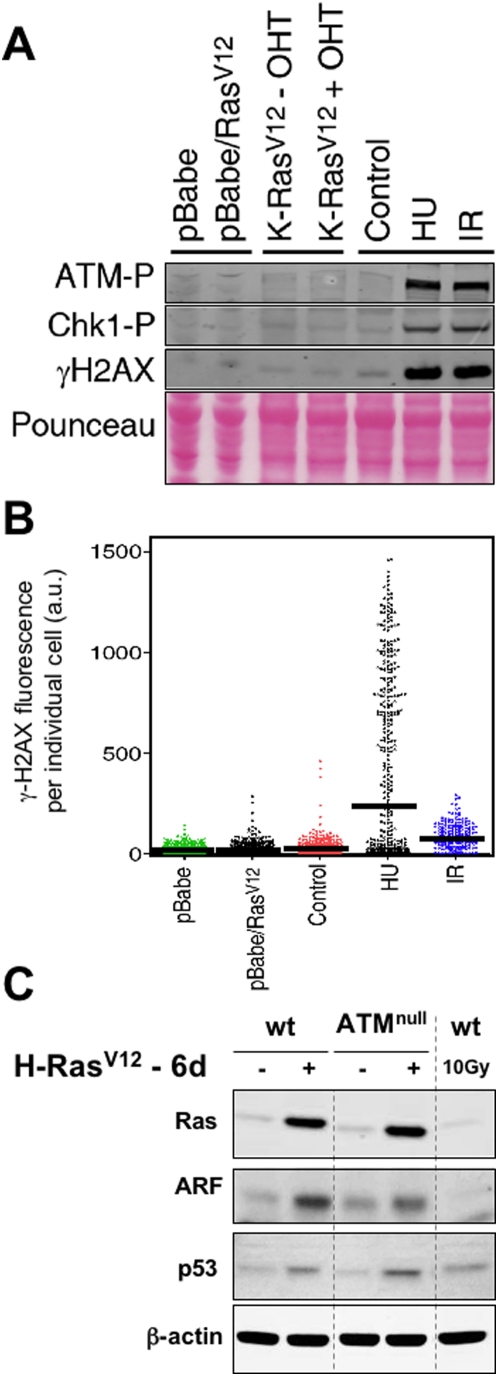
Lack of evidence for DNA damage signaling and limited role of Atm in Ras-induced senescence in murine fibroblasts. A. Immunoblots illustrating the phosphorylation status of Atm, Chk1 and H2AX under different conditions: infection of early passage primary MEFs with an empty vector or with H-*Ras*V12 expressing retrovirus (analyzed 6 days post-selection when cells were morphologically senescent), activation of an endogenous K-*Ras*V12 allele with 4-hydroxy-tamoxifen (OHT), and finally controls of DNA damage by replicative stress using hydroxyurea (HU 1 mM, 3 hrs) and by DNA breaks using ionizing radiation (IR 3 Gy, 45 min). B. Quantitative immunofluorescence of γH2AX in single cells from the same populations analysed in part A using high-throughput microscopy. The average intensity of the population is indicated with a bar. Note that upon replicative stress (HU) only the fraction of cells in S-phase activate the DDR, while upon irradiation (IR) the entire population activates the DDR. C. Immunoblots of the indicated proteins 6 days after selection of cells retrovirally transduced with H-*Ras*V12.

It has been firmly established in human fibroblasts that oncogenic Ras triggers DNA damage and activation of ATM [21]–[23]. To test this in murine fibroblasts, we overexpressed oncogenic H-Ras by retroviral transduction in wild-type primary mouse embryo fibroblasts (MEFs) and we also activated with 4-hydroxy-tamoxifen (OHT) an inducible endogenous allele of oncogenic K-Ras [31]. In the case of overexpressed oncogenic H-Ras, cells were analyzed 6 days post-infection when, as expected, cells had a clear senescent morphology. In the case of endogenous oncogenic K-Ras, cells continued proliferating, as previously reported [31] and in agreement with the concept that oncogenic signaling must reach a certain threshold before triggering senescence [29]. Interestingly, neither endogenous nor ectopic Ras signaling were able to activate a DNA damage response as judged by the levels of phosphorylated Atm, Chk1 or H2AX (Fig. 1A). To exclude the possibility that DNA damage signaling could occur in a small subpopulation of Ras-expressing MEFs, we used a sensitive automated immunofluorescence method that allows quantifying the signal intensity of phosphorylated-H2AX (γH2AX) in individual nuclei. Again, senescent cells overexpressing oncogenic H-Ras did not present evidence of an ongoing DNA damage response, not even in a minority of cells (Fig. 1B). These results suggest that murine fibroblasts undergo Ras-triggered oncogene-induced senescence in the absence of detectable DNA damage signaling. This implies a fundamental difference between human and murine fibroblasts with regard to their susceptibility to activate DNA damage signaling secondary to oncogenic signaling by Ras.

*Atm*-null fibroblasts undergo premature senescence due to their high levels of reactive oxygen species and DNA damage [32], [33], thus precluding an analysis of oncogene-induced senescence. Therefore, we restricted our analysis to the ability of oncogenic H-Ras to activate p53 in the absence of Atm. Interestingly, both Arf and p53 levels were increased in *Atm*-null MEFs upon overexpression of oncogenic H-Ras, in a manner indistinguishable from wild-type MEFs (Fig. 1C). Moreover, *Atm*-null MEFs were not permissive to H-RasV12-driven proliferation, either alone or in combination with the cooperating oncoprotein E1a (Supplementary Fig. S1). Although, as mentioned above, the lack of permissiveness to oncogene-driven proliferation could be due in part to the intrinsic premature senescence of *Atm*-null MEFs. Together, these results indicate that, in murine fibroblasts, oncogenic Ras signaling activates p53 independently of Atm.

### Atm is not necessary for senescence in K-RasV12-driven lung adenomas

Lung tumorigenesis induced by endogenous oncogenic K-*Ras* alleles faithfully recapitulates human lung adenocarcinoma [34], and presents two characteristics of relevance for this study, namely, tumorigenesis is suppressed by p53 [35], [36] and it is accompanied by a readily detectable senescence response at the pre-malignant tumor stages [37]. Therefore, this is a well-suited cancer model to evaluate *in vivo* the proposed role of *Atm* as a general barrier to cancer, including lung cancer [19], [20], through the activation of oncogene-induced senescence [21], [22]. Specifically, we used mice carrying the above-mentioned OHT-inducible endogenous K-*Ras*V12 allele [31]. The oncogenic allele is linked to a LacZ reporter that allows detection of those cells in which the oncogene has been activated by Cre-mediated excision upon OHT. For simplicity, we will refer to these mice as “K-*Ras*V12 mice”. In the following paragraphs, we describe separately the phenotype of *Atm*-heterozygous and *Atm*-nullizygous mice when combined with the K-*Ras*V12 allele activated by OHT at 1 month of age.

**Figure 2:**
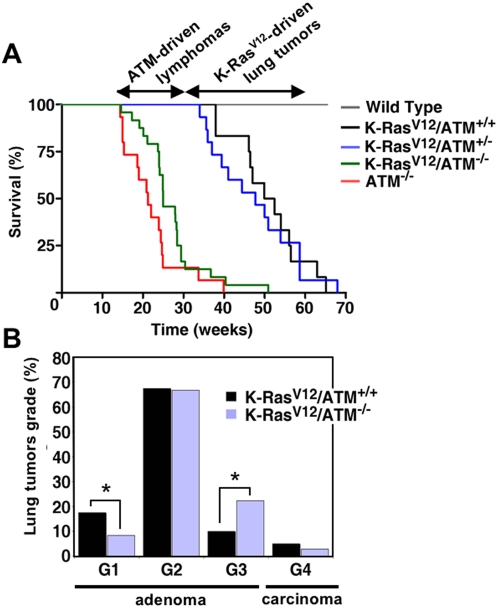
Limited role of Atm in Ras-driven murine lung tumorigenesis. A. Kaplan-Meier survival curves of the indicated mice (n = 15 mice for each group). The inducible K-*Ras*V12 allele was activated with OHT when the mice were 1 month old. B. Quantification of the different grades of lesions found in the lungs of K-*Ras*V12/Atm-wt and K-*Ras*V12/Atm-null mice (15 mice per group, and a total of n = 121 and n = 108 lesions, respectively). Asterisks indicate statistical significance of the Fisher's test *p*<0.05.

Regarding *Atm*-null mice, these mice succumb to thymic lymphomas at an early age [3]–[5] and we observed that the presence or absence of the K-*Ras*V12 allele did not affect survival in an *Atm*-null context (Fig. 2A). Examination of moribund *Atm*-null mice indicated that all of them, with or without K-*Ras*V12, developed thymic lymphomas with similar histopathological characteristics (not shown). All the thymic lymphomas (n = 5) examined from K-*Ras*V12*/Atm*-null animals were positive for LacZ (Supplementary Fig. S2; note that this staining is at pH 7 and does not produce cross-staining with senescence-associated β-galactosidase, which is measured at pH 6). The above is an interesting observation because expression of the K-*Ras*V12 oncogene was achieved in ∼8% of the cells of the thymus (quantified in scanned photographs; see example in Supplementary Fig. S2). These observations suggest that those thymocytes carrying the activated oncogene have a selective advantage compared to the non-oncogenic ones. We conclude that K-RasV12 has a modest impact on thymic lymphomagenesis driven by *Atm*-loss, providing a selective advantage to the tumoral cells, but without accelerating tumor onset.

In agreement with previous results [31], oncogenic activation of K-Ras in 1-month old mice led to lung carcinogenesis with full penetrance and death between 9 and 16 months of age. Unexpectedly, *Atm*-heterozygosity did not diminish significantly the survival of K-*Ras*V12 mice when compared with their control *Atm* wild-type mice (Fig. 2A; logrank test *p* = 0.79) and histopathological analyses confirmed that death was due to the presence of multiple lung tumors.

Despite the previous negative data using *Atm*-heterozygous mice, we reasoned that the role of Atm in K-RasV12-driven lung tumorigenesis could perhaps be revealed in mice completely deficient in *Atm*. For this, we sacrificed *Atm*-null and *Atm*-wt mice carrying the activated K-*Ras*V12 allele at 5 months of age (4 months post-activation with OHT), when most *Atm*-null mice had not developed yet frank lymphomas but lung tumors were already present. Tumors were counted and grouped in 4 grades, from adenomas (G1–G3) to adenocarcinomas (G4), according to the classification of Johnson *et al*. [35]. Quantification of the number of lesions indicated that the status of *Atm* had no significant impact on tumor incidence (*Atm*-wt: 8.1 lesions/mouse; *Atm*-null: 7.2 lesions/mouse; n = 15 per genotype). Interestingly, histopathological analysis of more than 100 lesions per genotype revealed that *Atm*-nullizygosity had a modest, albeit significant, effect on tumor progression (Fig. 2B). This effect was restricted to the pre-malignant stages with a ∼50% decrease in G1 adenomas and a ∼50% increase in G3 adenomas in *Atm*-null mice compared to their wt controls. At this time of analysis (5 months of age), no significant differences were observed in the incidence of malignant tumors (G4, adenocarcinoma) (Fig. 2B).

**Figure 3:**
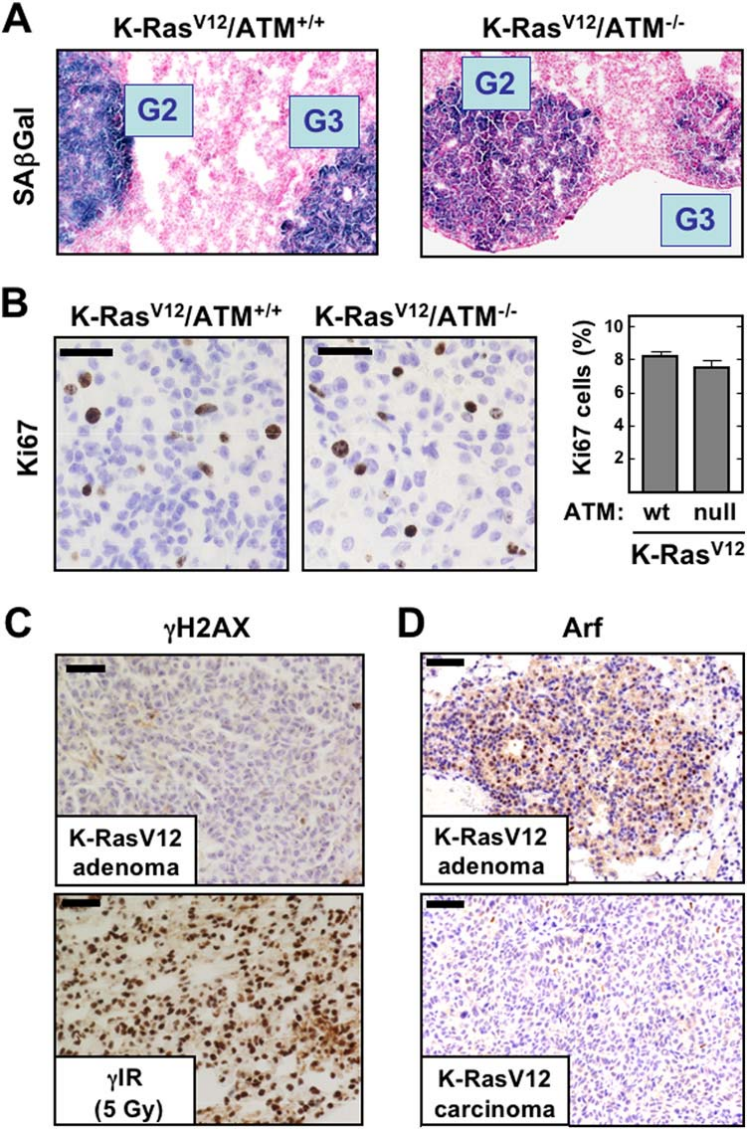
Lack of evidence for DNA damage signaling and limited role of Atm in senescent murine lung tumors. A. Representative stainings of senescence-associated β−galactosidase (SAβGal) in lung cryosections from K-*Ras*V12/Atm-wt and K-*Ras*V12/Atm-null mice. The grade of the tumor (G2 or G3) is indicated. See Supplementary Fig. S3 for panoramic views, including adenocarcinomas (G4, which are negative for SAβGal). B. Examples of Ki67 stainings of lung adenomas from the indicated mouse genotypes and quantification (adenomas n = 4 for each genotype). Values correspond to the average and standard deviation. C. Lack of detectable γH2AX staining in K-*Ras*V12-driven lung adenomas. The bottom panel shows a positive control of the lung from an irradiated wildt-type mouse (5 Gy, 45 min post-IR). D. Expression of Arf during K-*Ras*V12-driven lung tumorigenesis is specifically associated to the pre-malignant stages (adenomas, top), but not to the malignant ones (adenocarcinomas, bottom). Note that some cells in the adenoma are Arf-positive and others are Arf-negative, consistent with the concept that not all the tumor cells of these lesions are senescent, and indeed a fraction of cells are proliferating, as shown in part B.

Next, we wondered whether, despite the modest effect of *Atm*-deficiency on tumor progression, lung adenomas in *Atm*-null mice had lost the senescence response characteristic of these tumors [37]. Senescence was evaluated by the widely accepted marker senescence-associated β-galactosidase (SAβGal; [38]). Remarkably, *Atm*-null adenomas presented a senescence response comparable to *Atm*-wt adenomas (Fig. 3A; for whole lung panoramic views see Supplementary Fig. S3). As previously reported [37], adenocarcinomas were negative for SAβGal (see example in Supplementary Fig. S3). Moreover, *Atm*-null adenomas had the same proliferative index as *Atm*-wt adenomas (Fig. 3B), thus indicating that *Atm* is not a barrier for the proliferation of these pre-malignant lesions (apoptosis was essentially undetectable in these lesions, not shown). These negative data prompted us to evaluate whether the K-RasV12-driven adenomas had an ongoing DNA damage response, however, we were unable to detect γH2AX positive cells in the adenomas (Fig. 3C). Finally, as an additional control, induction of the senescence mediator Arf was readily detected in lung adenomas, but not in carcinomas (Fig. 3D), further reinforcing the idea that oncogene-induced senescence in this model occurs in the absence of DNA damage signaling.

Collectively, we conclude from the above data that senescence at the pre-malignant stages of K-RasV12-driven lung tumorigenesis is not associated to a detectable DNA damage response and it is independent of the status of *Atm*. Accordingly, the absence of *Atm* has a modest impact on the pre-malignant stages of this tumorigenesis model. It is conceivable that loss of function of Atm could increase aggressiveness at later stages of tumorigenesis.

### Limited role of Atm in protection from chemically-induced fibrosarcomas

**Figure 4:**
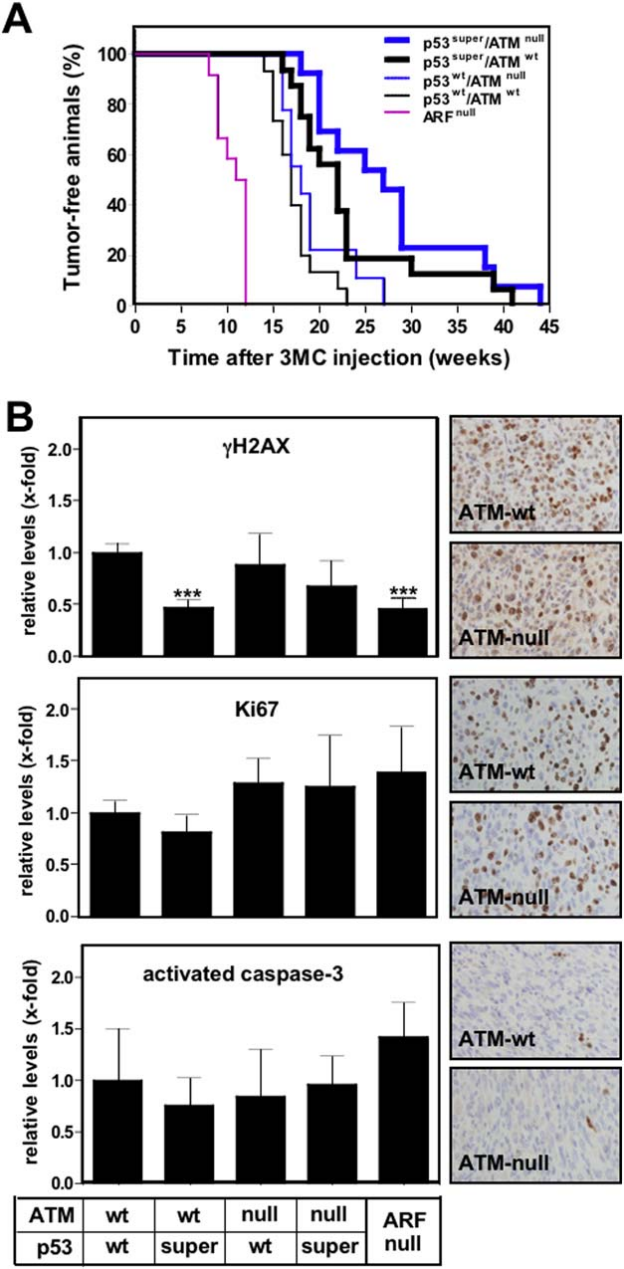
Limited role of Atm in p53-mediated tumor suppression and DNA damage response in chemically-induced fibrosarcomas. A. Mice of the indicated genotypes, wt (n = 15), *p53*-super (n = 16), *Atm*-null (n = 9), *p53*-super/*Atm*-null (n = 13) and *Arf*-null (n = 12), were injected intramuscularly with 3-methyl-cholanthrene (3MC) and tumour development was monitored. Kaplan-Meier tumour-free curves were obtained and statistical significant differences (logrank test) were found for wt vs. *p53*-super (*p*<0.005), *Atm*-null vs. *p53*-super/*Atm*-null (*p*<0.001) and *Arf*-null vs. wt (*p*<0.0001). No significant differences were found for wt vs. *Atm*-null (*p* = 0.11), or *p53*-super vs. *p53*-super/*Atm*-null (*p* = 0.18). B. Quantification of the persistent DNA damage response (γH2AX), proliferation (Ki67), and apoptosis (activated caspase-3) in 3MC-fibrosarcomas generated in mice of the indicated genotypes. Quantifications are relative to the 3MC-fibrosarcomas in wild-type mice. Values correspond to the average and standard deviation (n = 5 per genotype). Examples of the immunostainings are shown in the panels at the right.

To further evaluate the role of Atm in a cancer model initiated by DNA damage and under a strong control by p53-mediated tumor suppression, we performed chemical carcinogenesis with the DNA damaging agent 3-methyl-cholanthrene (3MC). This carcinogen generates fibrosarcomas when injected intramuscularly in mice, and tumors carry frequent oncogenic mutations in K-*Ras* or N-*Ras* [28], [39]–[41], together with a high incidence of *p53* inactivation by deletion or point mutation [41], [42]. Confirming the relevant role of p53 in the control of these tumors, we have previously shown that the latency of 3MC-fibrosarcomas is highly sensitive to the activity of p53. In particular, mice carrying three functional alleles of the *p53* gene (*p53*-super) have a 50% increase in *p53* gene dosage relative to wild-type mice (*p53*-wt), and this increase in p53 activity translates into a significant delay in the latency of 3MC-fibrosarcomas [43]. To evaluate the role of Atm in this *in vivo* assay of p53-mediated tumor suppression, we performed the appropriate crosses to compare *p53*-wt and *p53*-super in an *Atm*-null background. As validation of our genetic system, we observed that the short-term response of p53 to acute DNA damage (3h post-irradiation) was abrogated in the absence of *Atm*, regardless of the *p53* gene dose; and, in the presence of *Atm*, the levels of p53 and p21 were directly associated to the *p53* gene dose (Supplementary Fig. S4). However, unexpectedly, the status of *Atm* did not affect the latency of 3MC-fibrosarcomas neither in a *p53*-wt context nor in a *p53*-super context (Fig. 4A; statistical analyses indicated in the legend). The lack of effect of Atm is in contrast to the dramatic effect of Arf, whose deficiency completely abrogates p53-mediated tumor suppression in this cancer model without affecting the short-term response to DNA damage [28] (see also Fig. 4A). Tumors from all the genotypes (n = 5 per genotype) were characterized histologically and all of them showed a strong constitutive DNA damage response (∼50% of cells positive for γH2AX), a high mitotic index (∼50% of cells positive for Ki67), and very low apoptosis (<1% of cells positive for activated caspase-3) (Fig. 4B). Quantification of these markers indicated that their prevalence was not affected by the status of *Atm* (Fig. 4B). The presence of high levels of γH2AX in *Atm*-null tumors indicates that other kinases from the Atm family are responsible for the phosphorylation of H2AX, such as Atr or Dnapk. Of note, tumors arising in *Arf*-null mice or in *p53*-super mice showed a modest, albeit significant, decrease in the number of γH2AX-positive cells (Fig. 4B).

**Figure 5:**
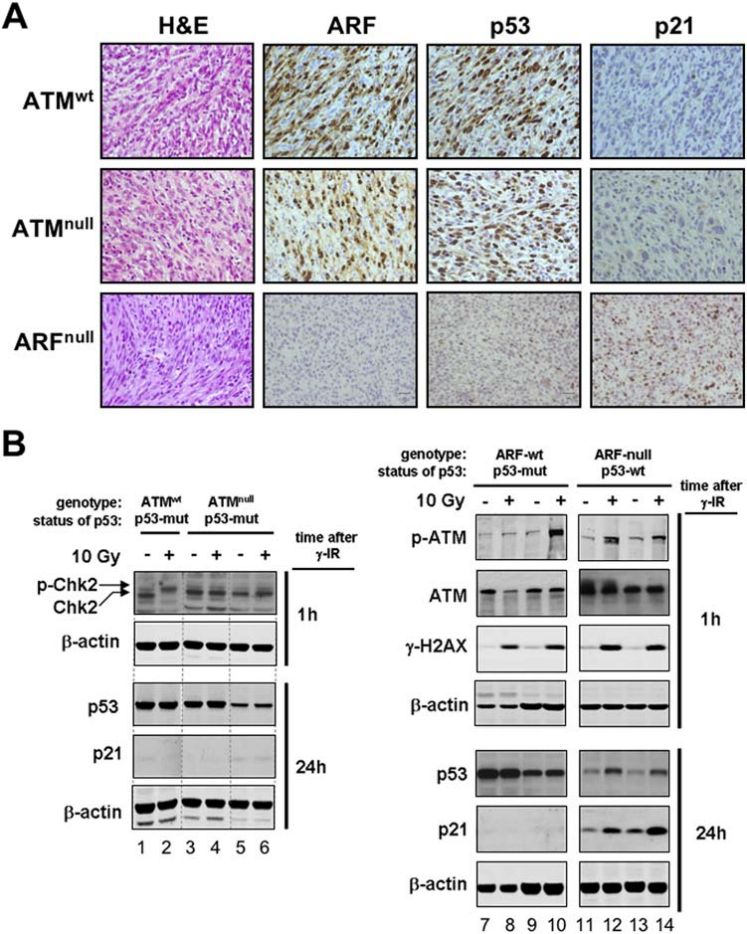
Absence of Atm does not relieve the selective pressure to inactivate p53 during chemical carcinogenesis. A. Representative images of 3MC-fibrosarcomas immunostained for Arf, p53 and p21 (see also Table 1). The upper and middle rows are representative of the large majority of fibrosarcomas developed in wild-type (upper) and *Atm*-null (middle) mice, which are consistent with a mutant p53 (*i.e.* strongly positive for p53 and negative for p21). The lower row is representative of the fibrosarcomas developed in *Arf*-null mice, which are consistent with a functional p53 (*i.e.* very weakly positive for p53 and positive for p21). B. Examples of cancer cell lines established from 3MC-fibrosarcomas (each line derives from an independent fibrosarcoma). The genotype of the mice where the 3MC-fibrosarcomas were generated is indicated, as well as, the status of p53 as determined by a nutlin-sensitivity assay (see Supplementary Figures S5 and S6). Cell lines were exposed to 10 Gy and protein extracts were obtained 1h and 24h after irradiation. The levels of the indicated proteins were determined by immunoblotting using β-actin as loading control.

We wondered whether *Atm*-nullizygosity, despite its lack of effect on tumor latency, could relieve the selective pressure to inactivate p53. For this, we examined the functional status of p53 in 3MC-fibrosarcomas and in cell lines derived from these tumors. Inactivation of p53 by point mutation generally renders p53 abnormally stable and readily detectable by immunohistochemistry, and results in undetectable levels of the p53-target p21. The large majority of 3MC-fibrosarcomas produced in *Atm*-wt mice (19/22 = 86%, after combining *p53*-wt and *p53*-super) had an immunohistochemical profile consistent a “p53 mutant” pattern (*i.e.* p53-strongly positive & p21-negative; Table 1, see example in Fig. 5A), thus confirming the strong pressure to inactivate p53 in this cancer model. Importantly, a similar situation was observed in the 3MC-fibrosarcomas generated in *Atm*-null mice, where the majority (14/18 = 78%) also had a “p53-mutant” pattern, including those generated in a *p53*-super context (Table 1 and Fig. 5A). In contrast to this and in agreement with our previous data [44], tumors generated in *Arf*-null mice presented a “p53 functional” staining pattern (*i.e.* p53-weakly positive & p21- positive; Table 1, Fig. 5A and see Supplementary Fig. S5 for a nutlin-sensitivity assay), thus confirming that *Arf*-deficiency, in contrast to *Atm*-deficiency, completely relieves the pressure to inactivate *p53*.

**Table 1:** Immunohistochemical analysis of 3MC-fibrosarcomas.

Genotype	ARF positive	p53 positive (mutant p53) a	p21 positive (active p53) a
***Atm*** **-wt;** ***p53-*** **wt**	**10/10**	**9/10**	1/10
***Atm*** **-wt;** ***p53*** **-super**	**12/12**	**10/12**	1/12
***Atm*** **-null;** ***p53*** **-wt**	**7/7**	**7/7**	0/7
***Atm*** **-null;** ***p53*** **-super**	**8/11** b	**7/11**	3/11
***Arf*** **-null**	n.a.	1/9	7/9

a Most tumors strongly positive for p53, lacked p21 expression (reflecting a non-functional mutant p53); and, tumors negative or weakly positive for p53, had detectable p21 (reflecting a functional p53)

b The few tumors that lacked Arf were negative or weakly positive for p53 (reflecting a functional p53)

To further substantiate the above observations, we examined the functionality of p53. The cancer cell lines derived from wt or *Atm*-null mice had constitutively high levels of p53 and essentially undetectable levels of p21 both before and after ionizing radiation (Fig. 5B, lanes 1–10), compatible with a mutant p53 status. We wondered whether the 3MC-fibrosarcomas produced in wt mice retained a normal response to DNA damage. As shown in Fig. 5B, the phosphorylation of Chk2, Atm and H2AX shortly after ionizing radiation was normal in representative lines from wt mice (lanes 1,2,7–10) but, as expected, Chk2 phosphorylation was absent in the fibrosarcomas from *Atm*-null mice (lanes 3–6). Therefore, 3MC-fibrosarcomas derived from wild-type mice retain an apparently functional Atm and DNA damage response, and *Atm*-deficiency does not relieve the pressure to inactivate p53. The cancer cell lines derived from *Arf*-null mice also had a normal DNA damage response (Fig. 5B, lanes 11–14), but in sharp contrast with those fibrosarcomas from wt or *Atm*-null mice, *Arf*-null fibrosarcomas conserved a functional p53/p21 response to DNA damage (lanes 11–14).

Finally, to seek evidence for an impact of *Atm*-deficiency in this cancer model, we examined the level of genomic instability in the different 3MC-fibrosarcoma cell lines described above. Consistent with the well-established role of p53 in genomic stability, those fibrosarcomas lacking functional p53 had high levels of chromosomal fusions (Table 2). To dissociate the impact of p53 and Atm on genomic stability, we grouped the *Atm*-null cancer cell lines into those with mutant p53 and those with functional p53 (treatment with the MDM2 inhibitor nutlin confirmed the presence or absence of functional p53, see Supplementary Fig. S6). Interestingly, cancer cells deficient in Atm, but not in p53, had significantly higher levels of chromosomal instability, particularly fusions and aneuploidies, than the comparable *Arf-*null;p53-functional cells (Table 2). In summary, these observations indicate that the absence of *Atm* increases genomic instability, but it does not relieve the pressure to inactivate p53 during cancer development.

**Table 2:** Chromosomal instability in 3MC-fibrosarcoma cell lines.

Genotype of mouse	Status of p53 a	Fusions (% of chromosomes)	Aneuploidies (% of metaphases) c	Breaks (% of chromosomes)	Metaphases analyzed (cell lines analyzed)
***Atm*** **-wt**	**mutant**	**1.2±2.4**	**56±20**	**1.4±1.8**	294 (5)
***Atm*** **-null**	**mutant**	**2.4±12.0**	**85±22**	**14.0±66.0**	111 (2)
***Atm*** **-null** b	**functional**	**1.5±7.5** d	**74±34** d	**1.9±7.5**	109 (2)
***Arf*** **-null**	functional	0.09±0.41^d^	31±13^d^	1.0±6.0	287 (5)

a The functional status of p53 in the fibrosarcoma cell lines was determined by examining the levels of p53 and p21 upon γIR, both by immunohistochemistry (Fig. 5A) and immunoblotting (Fig. 5B), as well as, by their susceptibility to cell cycle arrest upon treatment with nutlin (Supplementary Fig. S5 and S6).

b These *Atm*-null lines with functional p53 correspond to a minority of the 3MC-fibrosarcomas produced in *Atm*-null mice, which by large have mutant p53 (see Table 1 and Fig. 5B). Only two lines were obtained that retained functional p53 (Supplementary Fig. S6): one derived from an *Atm*-null;*p53*-super mouse and the other one from an *Atm*-null;*p53*-wt mouse.

c Aneuploidies correspond to metaphasases with chromosome numbers different from 40±1 or 80±2.

d Statistical comparison (t-test) between *Atm*-null(p53-functional) and *Arf*-null(p53-functional): metaphases with fusions, *p* = 0.04; aneuploid cell lines, *p* = 0.06.

Collectively, we conclude that Atm plays a minor role in 3MC carcinogenesis. In the absence of *Atm*, genomic instability is favored, yet neither the DNA damage response nor p53-mediated tumor suppression are affected.

## Discussion

The role of ATM in tumor suppression is solidly established and attributed, at least in part, to its well-known function in DNA damage repair. In addition to its function in guarding the genome, ATM has been recently proposed to act as a general tumor suppressor through its ability to activate p53 in response to oncogenic stress and the subsequent induction of oncogene-induced cellular senescence [17], [18]. This model is mainly based on human cultured fibroblasts where ATM plays an essential role in oncogene-induced senescence [21]–[23]. In this work, we have attempted to find *in vivo* supporting evidence for the role of ATM in p53-dependent tumor suppression and in oncogene-induced senescence using murine experimental systems, namely, Ras-induced senescence in mouse embryo fibroblasts (MEFs), Ras-induced senescence during lung tumorigenesis, and chemically-induced fibrosarcomas.

A first unexpected observation was the absence of a detectable DNA damage response upon ectopic overexpression of oncogenic H-Ras in MEFs, under conditions that resulted in upregulation of Arf, stabilization of p53, and acquisition of a fully senescent morphology. Given the absence of a detectable DNA damage response elicited by oncogenic H-Ras, it was not surprising that the stabilization of p53 in response to the oncogene was not affected by the absence of Atm. Conceptually similar observations were made in a more physiological model of oncogene-induced senescence in which an inducible endogenous K-*Ras*V12 allele drives the development of adenomas (pre-malignant) and adenocarcinomas (malignant), being characterized the adenomas by a strong senescence response [37]. Again, as in the case of MEFs, we could not detect evidence of persistent DNA damage signaling in the K-*Ras*V12-driven adenomas, which were invariably senescent as shown in [37] (Fig. 3C). Consistently with this, *Atm* deficiency did not ablate the senescent response characteristically present in lung adenomas. Interestingly, however, the absence of *Atm* had a modest, albeit significant, effect in the degree of progression of adenomas. In contrast to these data, ablation of *Arf* strongly accelerates lung tumorigenesis driven by oncogenic K-*Ras*V12 [45], and it eliminates oncogenic Ras-induced senescence during mammary tumorigenesis [29], [30]. Together, these results indicate that oncogene-induced senescence in murine fibroblasts and lung epithelial cells is not associated to an ongoing DNA damage response and, therefore, activation of p53 and establishment of senescence occur independently of Atm.

Based on the above data, we focused on a murine cancer model initiated by a DNA damage agent (3-methyl-cholanthrene or 3MC), known to be highly sensitive to the activity of p53 [43], and accompanied by a persistent DNA damage response (see Fig. 4B). However, the absence of *Atm* did not impact on the kinetics of tumor development, even under conditions able to detect a 1.5-fold difference in p53 activity. Histopathological analyses of these tumors indicated that the absence of *Atm* did not decrease the persistent DNA damage response, which must be signaled by other kinases of the Atm family, such as Atr or Dnapk. Importantly, the absence of Atm did not relieve the strong selective pressure to inactivate p53, further confirming that the tumor suppressor activity of p53 in this cancer model is not regulated by Atm. These results are in agreement with previous reports that also failed to detect an impact of Atm on p53-mediated tumor suppression in murine brain and skin cancers [46], [47]. The absence of effect of Atm on p53-dependent tumor suppression is in sharp contrast with a number of reports where the absence of Arf essentially eliminates p53-mediated tumor suppression and completely alleviates the selective pressure to inactivate p53 in lymphomas, sarcomas and skin tumors [26]–[28], [48], [49]. Moreover, the 3MC-fibrosarcomas originated in *Arf*-null mice retained a functional response to DNA damage, as judged by the phosphorylation of Atm and H2AX, as well as, by the stabilization of p53 and the induction of p21 upon irradiation (Fig. 5B). In summary, in this cancer model initiated by a DNA damage agent and associated to persistent DNA damage, the absence of *Atm* does not have a detectable effect on DNA damage signaling or on p53-dependent tumor suppression. Finally, and interestingly, we found that *Atm*-null fibrosarcomas were characterized by high levels of chromosomal instability (Table 2), thus testifying to the known role of Atm in genome stability.

It is critically important to emphasize that our data do not negate a tumor suppressor role for ATM, which is solidly established both in human and mice (see Introduction), but question the generality of the model proposed for ATM as a critical mediator of p53-activation and senescence in response to oncogenic signaling [17], [18]. This model is mainly based on the behavior of human fibroblasts *in vitro* and it does not seem to apply to the three murine experimental systems analyzed here, namely, oncogene-induced senescence in fibroblasts and during lung tumorigenesis, and p53-mediated tumor suppression during carcinogenesis initiated by a DNA damage agent. By large, the molecular biology of human and murine cancer seem to follow the same mechanistic paradigms, however, it is conceivable that ATM is an exceptional case playing a major tumor suppression role in human cancer, but not in murine cancer.

## Materials and Methods

### Ethics statement

Mice were treated in accordance with the Spanish Laws and the Guidelines for Humane Endpoints for Animals Used in Biomedical Research.

### Cells and mice

K-*Ras*V12;*Atm*-null mice were obtained by crossing transgenic mice carrying a tamoxifen-inducible Cre and a Cre-inducible K-*Ras*V12 endogeneous allele [31] with *Atm*-null mice [4]. Activation of the Cre-inducible K-*Ras*V12 was performed in 1-month old mice by injection of tamoxifen as previously described [31]. *Arf*-null mice [50] and *p53*-null mice [51] were previously described. Mice were housed under standard conditions at the serum-pathogen free facility of the Spanish National Cancer Research Centre (CNIO). Upon signs of morbidity or when required, mice were euthanized. Mouse embryo fibroblasts (MEFs) were obtained from E13.5 embryos as previously described [52] and grown in Dulbecco's Minimum Essential Media (DMEM, Invitrogen, Grand Island, NY, USA) supplemented with 10% FBS (Hyclone, Logan, UT). MEFs were grown in 3% oxygen to minimize premature senescence of *Atm*-deficient cells. In all experiments with MEFs, low passage (less than 3 passages) MEFs were used.

### Oncogene-induced senescence in MEFs

Cells were retrovirally transduced following standard procedures using constructs based on the vector pBabe-puro (Fig. 1A) or pLPC-puro (Fig. 1C and Suppl. Fig. S1) and expressing oncogenic Ras (H-*Ras*V12) or E1a/IRES/H-*Ras*V12 [53]. Infected cells were selected with 1.5 µg/ml puromycin for 2–3 days as described [54]. Cell extracts were prepared 6 days post-selection. MEFs carrying a tamoxifen-inducible K-*Ras*V12 allele were also used [31]. Quantification of DNA damage by quantitative immunofluorescence of single cells was performed as described [55]. Briefly, cells were grown on μCLEAR-bottom 96-well dishes (Greiner Bio-One) and analyzed on a BD Pathway 855 BioImager (Beckton Dickinson). Image analysis was performed with the AttoVision software (Beckton Dickinson). All the images for quantitative analyses were acquired under nonsaturating exposure conditions.

### Tumor induction and establishment of fibrosarcoma cell lines

For fibrosarcomagenesis, 2–4 mo old animals were injected intramuscularly in one of the rear legs with 1 mg of 3-methyl-cholanthrene (Sigma) dissolved in corn oil, and tumor development was followed, as previously described [43]. Mouse fibrosarcoma cell lines were obtained from tumors as previously described [43]. Briefly, the tumor mass was rinsed in phosphate-buffered saline (PBS) with antibiotics and then minced with razor blades into small pieces that were placed in 100-mm diameter dishes in the presence of DMEM/10% FBS. Cell lines were established from tumor outgrowths after four to six passages. Cells were plated in 3.5-cm diameter plates and, the following day, were irradiated with 10 Gy at 1.94 Gy/min in a Shepherd Mark 1–30 irradiator.

### Metaphase analyses

Exponentially growing cells were incubated with 0.1 µg/ml colcemide (Gibco, Invitrogen, Paisley, UK) for 2 h at 37°C and then fixed in methanol:acetic acid (3∶1). Metaphases were spread in acetic acid 45%. Chromosome number, fragments and fusions were analyzed in at least 50 metaphases per cell line.

### Immunoblots and immunohistochemistry

Whole-cell protein extracts were obtained using RIPA buffer. For immunoblotting, we used the following primary antibodies: anti-p53 (NCL-p53-CM-5p, Novocastra; and 1C12, Cell Signaling Technology), anti-p21 (p21-C-19-G, Santa Cruz Biotechnology), anti-total-Atm (NB100-220, Novus Bio), anti-phospho-Ser1981-Atm (Atmp1981, 200-301-400, Rockland Biochemicals), anti H-Ras (clone 18, BD Biosciences), anti-Arf (Ab80-100, Abcam; and 5-C3-1, Santa Cruz Biotechnology), anti-Chk2 (05-649, clone 7, Upstate), anti-phospho-Chk1 (2341, Cell Signaling Technology), anti-γH2AX (05-636, Millipore), and anti-β-actin (clone AC-15, Sigma). Protein levels were visualized after incubation with the appropriate secondary antibodies conjugated with HRP followed by detection with ECL Plus (Amersham) or conjugated with fluorescein followed by detection with Oddysey (Li Cor Biosiences).

For immunohistochemistry, formalin-fixed paraffin-embedded tumor samples were incubated with the following antibodies: anti-Ki67 (TEC-3, DAKO), anti-γH2AX (clone JBW301, Upstate), anti-Caspase 3 active (R&D Systems), anti-Arf (5-C3-1, Santa Cruz Biotechnology), anti-p53 (NCL-p53-CM-5p, Novocastra), and anti-p21 (p21-C-19-G, Santa Cruz Biotechnology). For quantification, 10 high power fields (40×) were randomly chosen under the microscope and the total number of positive cells was counted for each tumor.

#### SA-β-Galactosidase activity

SA-β-Galactosidase activity was evaluated using the “Senescence-βGal Staining Kit” (Cell Signaling Technology) following the manufacturer's instructions in OCT-embedded lung sections. Stained slides were subsequently stained with nuclear fast red.

## Supporting Information

Figure S1 Atm-deficiency does not render MEFs permissive to H-RasV12-driven proliferation A. Primary mouse embryo fibroblasts (MEFs) of the indicated genotypes were retrovirally transduced with H-RasV12 and 2000 cells were seeded in 10-cm diameter plates. After 2 weeks, cells were fixed and stained and colonies were counted. All incubations were done in low oxygen (3%). Top, quantification; bottom, representative plates. B. Same as in A but after retroviral transduction of cells with oncoviral protein E1a and H-RasV12.
(0.34 MB TIF)

Figure S2 Expression of K-RasV12 provides a selective advantage for T-lymphomagenesis in Atm-null mice. Left, representative LacZ staining of the thymus 2 days after activation of the K-RasV12 allele by tamoxifen (the oncogenic allele is linked with an IRES to LacZ). Quantification indicates that approximately 8% of the cells are LacZ-positive. Right, representative image of a thymic lymphoma from a K-RasV12;Atm-null mouse. A total of 5 lymphomas were analyzed and all of them were strongly positive for LacZ.
(0.20 MB TIF)

Figure S3 In vivo senescence in K-RasV12-driven lung adenomas is not affected by the status of Atm. Complete lung sections at low magnification stained with senescence-associated β-galactosidase (SAβGal) and nuclear fast red. Slides were examined blindly by an expert pathologist, Dr. Marta Cañamero (CNIO), who determined the grade of the tumors. All the adenomas (grades G1 to G3) were positive for SAβGal. The lung in the right side contains an adenocarcinoma that is shown at high magnification below. Adenocarcinomas (grade 4) presented a very weak SAβGal staining.
(0.43 MB TIF)

Figure S4 The short-term response of the lung to DNA damage is eliminated in the absence of Atm and it is enhanced in the presence of an extra allele of p53. Mice of the indicated genotypes were irradiated and protein extracts were prepared from their lungs 3 h post-IR. The antibody used for phospho-Ser18-p53 was from Cell Signaling (#9284S).
(0.12 MB TIF)

Figure S5 Arf-deficiency relieves the selective pressure to inactivate p53 in 3MC-fibrosarcomas (nutlin-sensitivity assay). Fibrosarcoma cell lines obtained from the wt or Arf-null mice were treated with 10 µM of the active enantiomer of nutlin (nutlin-3a, N3a) or with the inactive enantiomer (nutlin-3b, N3b). Nutlin activates p53 by inhibiting MDM2. After 48 h, cells were fixed and the proportion of S-phase cells was determined by flow cytometry. All the fibrosarcoma cell lines derived from wt mice did not respond to N3b, thus indicating that p53 was not functional; in contrast, all but one cell lines derived from Arf-null mice responded to N3b by strongly decreasing proliferation.
(0.07 MB TIF)

Figure S6 Identification of Atm-null fibrosarcoma cell lines retaining functional p53 (nutlin-sensitivity assay). The large majority of 3MC-fibrosarcomas from Atm-null mice lacked functional p53 (see Table 1), however, two Atm-null cell lines were identified that retained a functional p53. These cell lines, #2 and #3 in the figure, responded to 10 µM nutlin (racemic mixture) by undergoing cell cycle arrest as measured by flow cytometry 48 h after treatment.
(0.06 MB TIF)
