# Correction: Limited Role of Murine ATM in Oncogene-Induced Senescence and p53-Dependent Tumor Suppression

**DOI:** 10.1371/journal.pone.0298441

**Published:** 2024-02-01

**Authors:** Alejo Efeyan, Matilde Murga, Barbara Martinez-Pastor, Ana Ortega-Molina, Rebeca Soria, Manuel Collado, Oscar Fernandez-Capetillo, Manuel Serrano

In Fig 1C [1], the dashed lines indicated splicing, but this was not clarified in the legend. In the left panel of Fig 5B, one dashed line (between lanes 2–3) indicated splicing, and the other dashed line (between lanes 4–5) did not indicate splicing. The legends have been amended to clarify the meaning of the dashed lines in each figure.

The available original data underlying Figs 1C and 5B are provided in S1 and S2 Files. Spliced images presented together were derived from the same blots.

The primary data to support all results in the article and Supporting Information files are available from the corresponding author except for the original image underlying the 24h β-actin loading control in the left panel of Fig 5B, which is no longer available.

**Fig 1:**
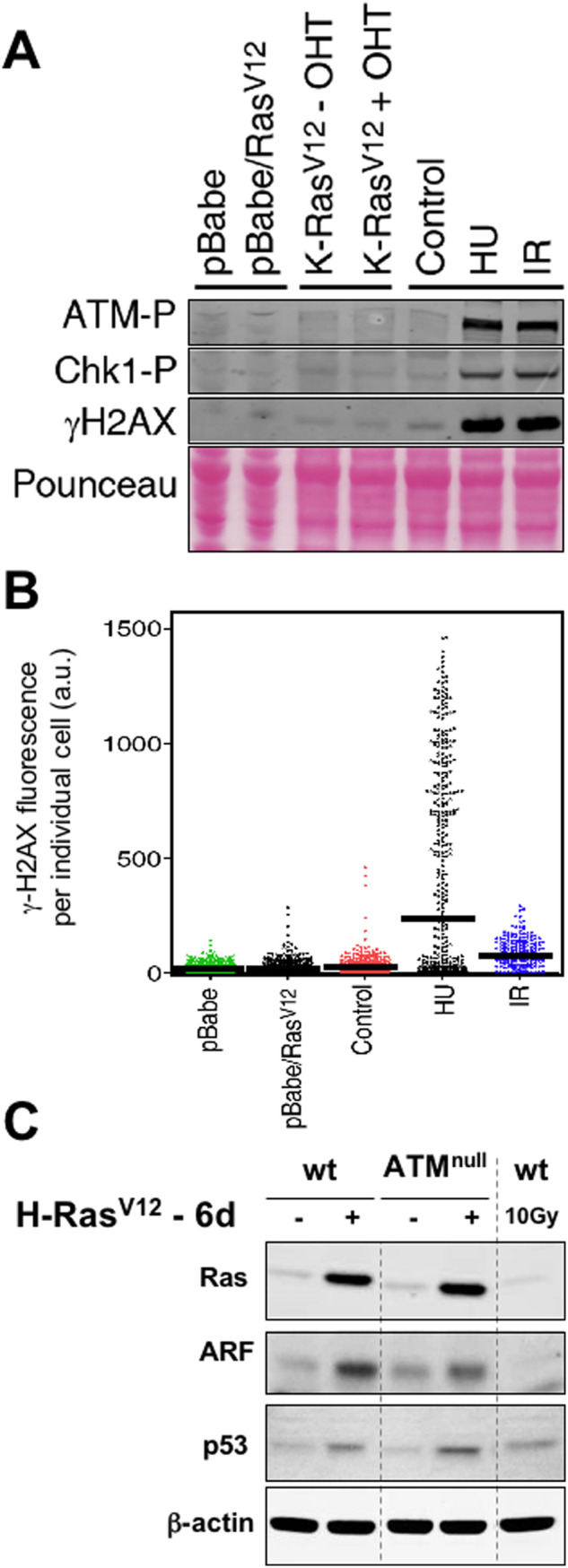
Lack of evidence for DNA damage signaling and limited role of Atm in Ras-induced senescence in murine fibroblasts. A. Immunoblots illustrating the phosphorylation status of Atm, Chk1 and H2AX under different conditions: infection of early passage primary MEFs with an empty vector or with H-*Ras*V12 expressing retrovirus (analyzed 6 days post-selection when cells were morphologically senescent), activation of an endogenous K-*Ras*V12 allele with 4-hydroxy-tamoxifen (OHT), and finally controls of DNA damage by replicative stress using hydroxyurea (HU 1 mM, 3 hrs) and by DNA breaks using ionizing radiation (IR 3 Gy, 45 min). B. Quantitative immunofluorescence of γH2AX in single cells from the same populations analysed in part A using high-throughput microscopy. The average intensity of the population is indicated with a bar. Note that upon replicative stress (HU) only the fraction of cells in S-phase activate the DDR, while upon irradiation (IR) the entire population activates the DDR. C. Immunoblots of the indicated proteins 6 days after selection of cells retrovirally transduced with H-*Ras*V12. Western blots were spliced at the positions indicated by dashed lines.

**Fig 5:**
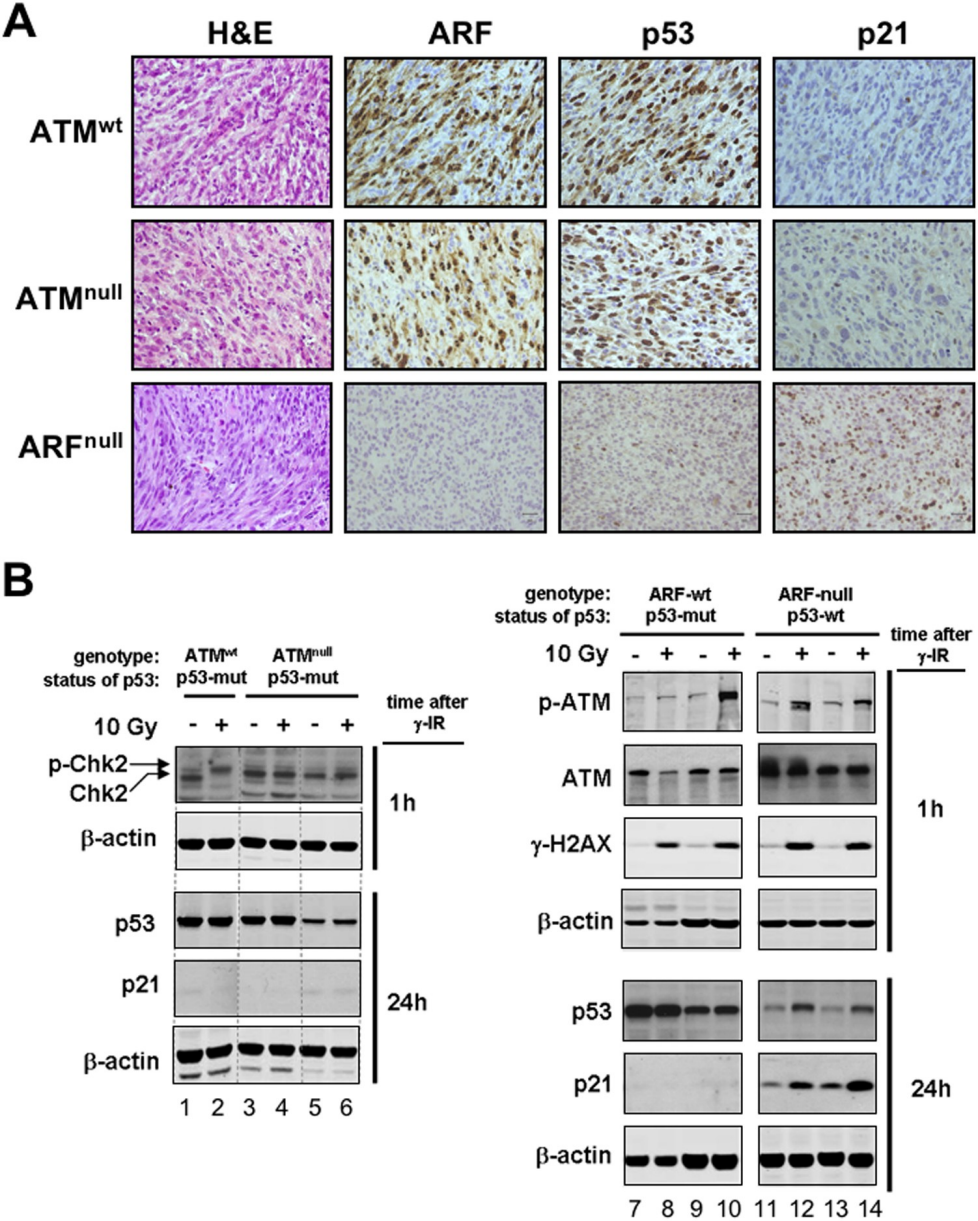
Absence of Atm does not relieve the selective pressure to inactivate p53 during chemical carcinogenesis. A. Representative images of 3MC-fibrosarcomas immunostained for Arf, p53 and p21 (see also Table 1). The upper and middle rows are representative of the large majority of fibrosarcomas developed in wild-type (upper) and *Atm*-null (middle) mice, which are consistent with a mutant p53 (*i*.*e*. strongly positive for p53 and negative for p21). The lower row is representative of the fibrosarcomas developed in *Arf*-null mice, which are consistent with a functional p53 (*i*.*e*. very weakly positive for p53 and positive for p21). B. Examples of cancer cell lines established from 3MC-fibrosarcomas (each line derives from an independent fibrosarcoma). The genotype of the mice where the 3MC-fibrosarcomas were generated is indicated, as well as, the status of p53 as determined by a nutlin-sensitivity assay (see Supplementary Figs S5 and S6). Cell lines were exposed to 10 Gy and protein extracts were obtained 1h and 24h after irradiation. The levels of the indicated proteins were determined by immunoblotting using β-actin as loading control. In the left-hand panel, western blots were spliced between lanes 2 and 3. The dashed line between lanes 4 and 5 does not indicate splicing.

## Supporting information

S1 File Annotated blots underlying Fig 1C. (PPTX)

S2 File Annotated blots underlying Fig 5B, except 24h β-actin (left panel). (PPTX)
