# Supplementary figures and images for: Limited Role of Murine ATM in Oncogene-Induced Senescence and p53-Dependent Tumor Suppression
Source: PLoS One. 2009 May 7;4(5):e5475. doi: 10.1371/journal.pone.0005475 (PMC2675057; doi:10.1371/journal.pone.0005475)

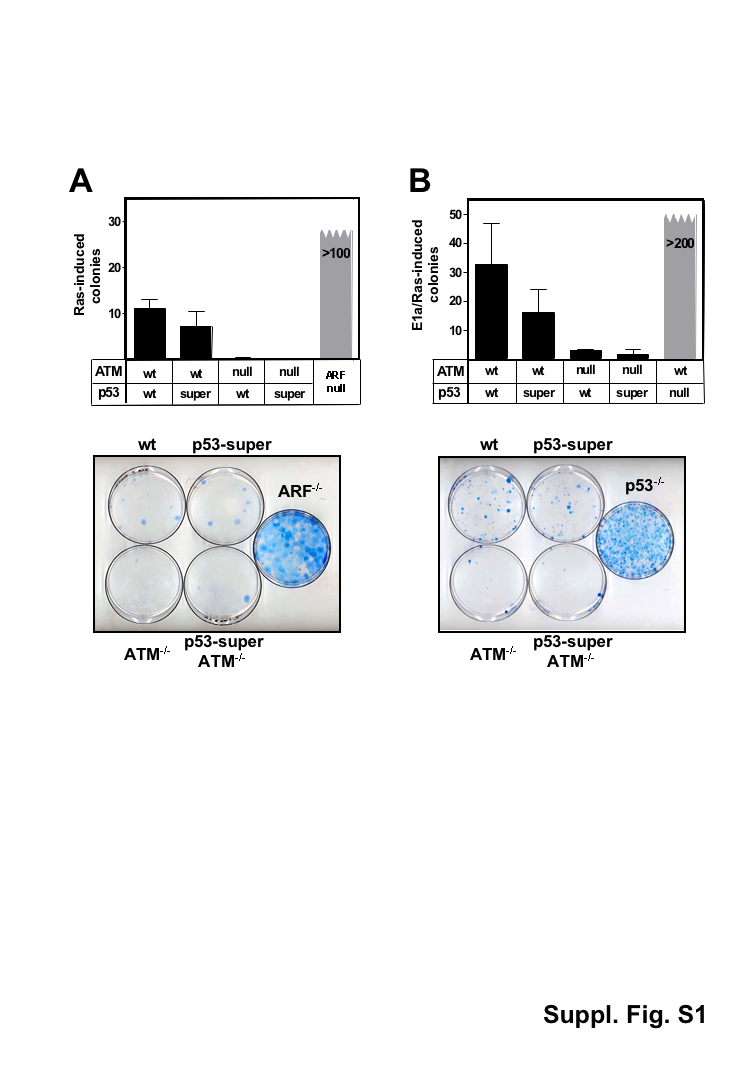

Supplement: Figure S1 — Atm-deficiency does not render MEFs permissive to H-RasV12-driven proliferation A. Primary mouse embryo fibroblasts (MEFs) of the indicated genotypes were retrovirally transduced with H-RasV12 and 2000 cells were seeded in 10-cm diameter plates. After 2 weeks, cells were fixed and stained and colonies were counted. All incubations were done in low oxygen (3%). Top, quantification; bottom, representative plates. B. Same as in A but after retroviral transduction of cells with oncoviral protein E1a and H-RasV12. (0.34 MB TIF) [file pone.0005475.s001.tif]

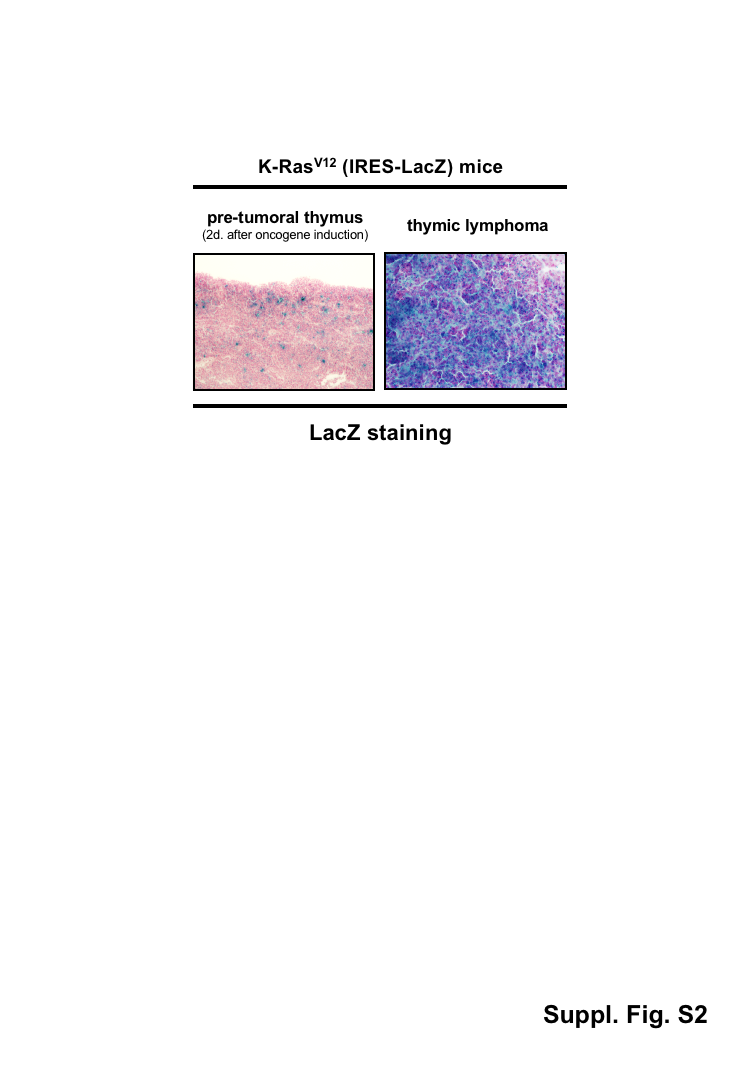

Supplement: Figure S2 — Expression of K-RasV12 provides a selective advantage for T-lymphomagenesis in Atm-null mice. Left, representative LacZ staining of the thymus 2 days after activation of the K-RasV12 allele by tamoxifen (the oncogenic allele is linked with an IRES to LacZ). Quantification indicates that approximately 8% of the cells are LacZ-positive. Right, representative image of a thymic lymphoma from a K-RasV12;Atm-null mouse. A total of 5 lymphomas were analyzed and all of them were strongly positive for LacZ. (0.20 MB TIF) [file pone.0005475.s002.tif]

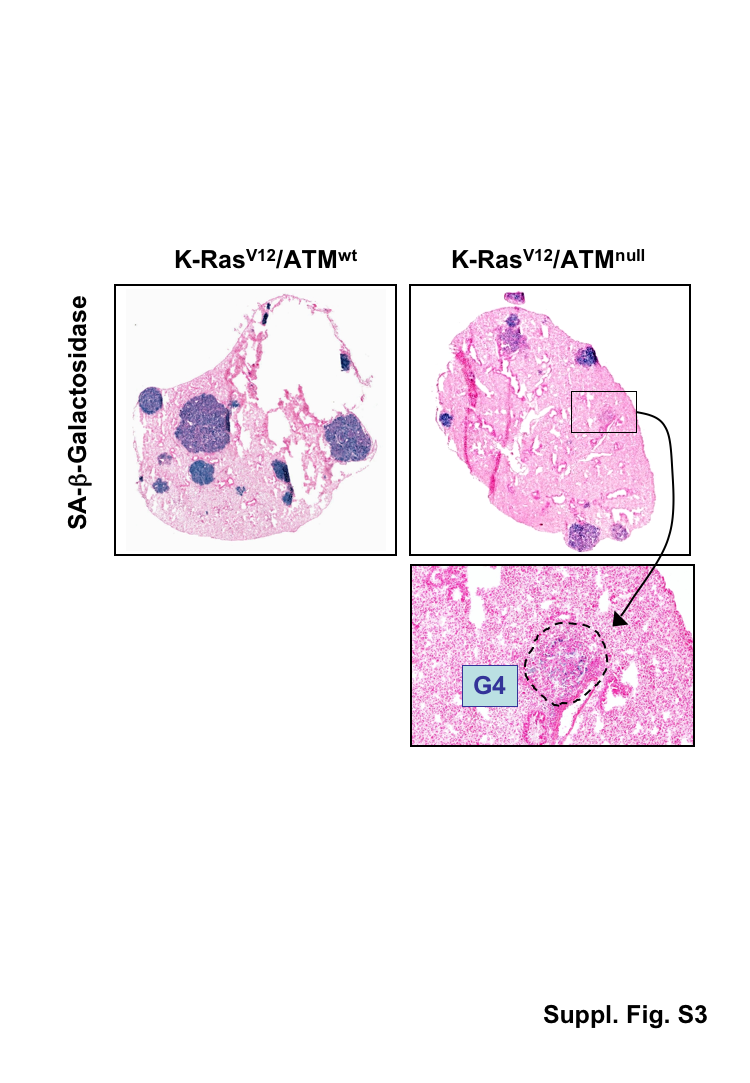

Supplement: Figure S3 — In vivo senescence in K-RasV12-driven lung adenomas is not affected by the status of Atm. Complete lung sections at low magnification stained with senescence-associated β-galactosidase (SAβGal) and nuclear fast red. Slides were examined blindly by an expert pathologist, Dr. Marta Cañamero (CNIO), who determined the grade of the tumors. All the adenomas (grades G1 to G3) were positive for SAβGal. The lung in the right side contains an adenocarcinoma that is shown at high magnification below. Adenocarcinomas (grade 4) presented a very weak SAβGal staining. (0.43 MB TIF) [file pone.0005475.s003.tif]

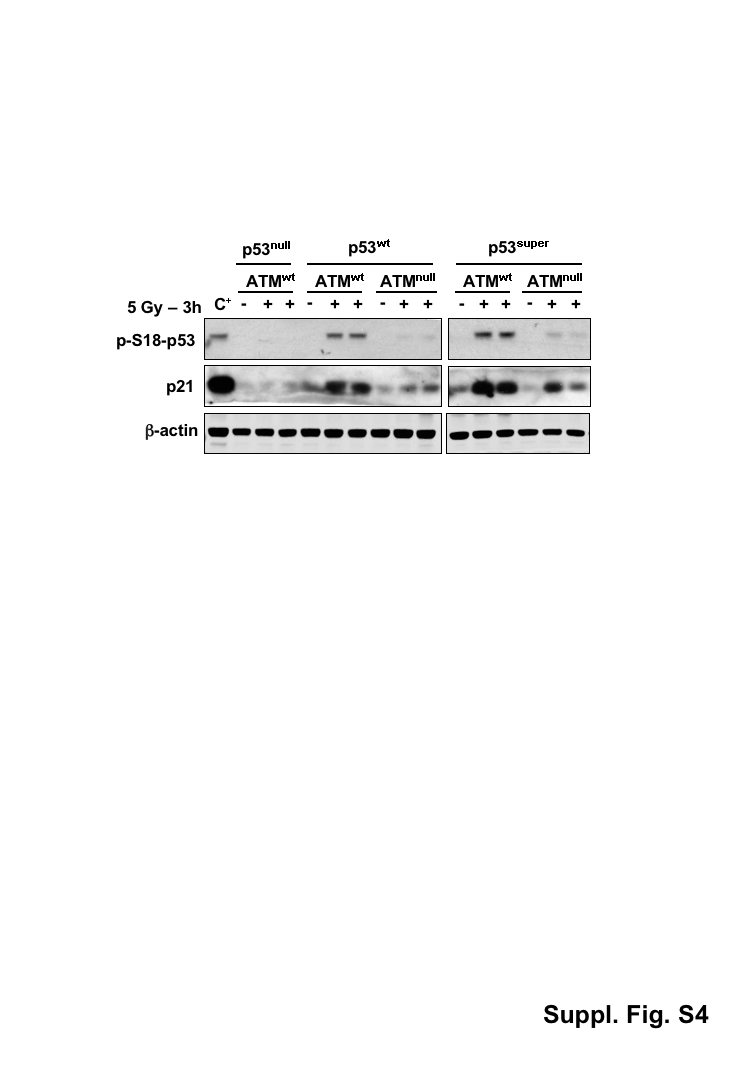

Supplement: Figure S4 — The short-term response of the lung to DNA damage is eliminated in the absence of Atm and it is enhanced in the presence of an extra allele of p53. Mice of the indicated genotypes were irradiated and protein extracts were prepared from their lungs 3 h post-IR. The antibody used for phospho-Ser18-p53 was from Cell Signaling (#9284S). (0.12 MB TIF) [file pone.0005475.s004.tif]

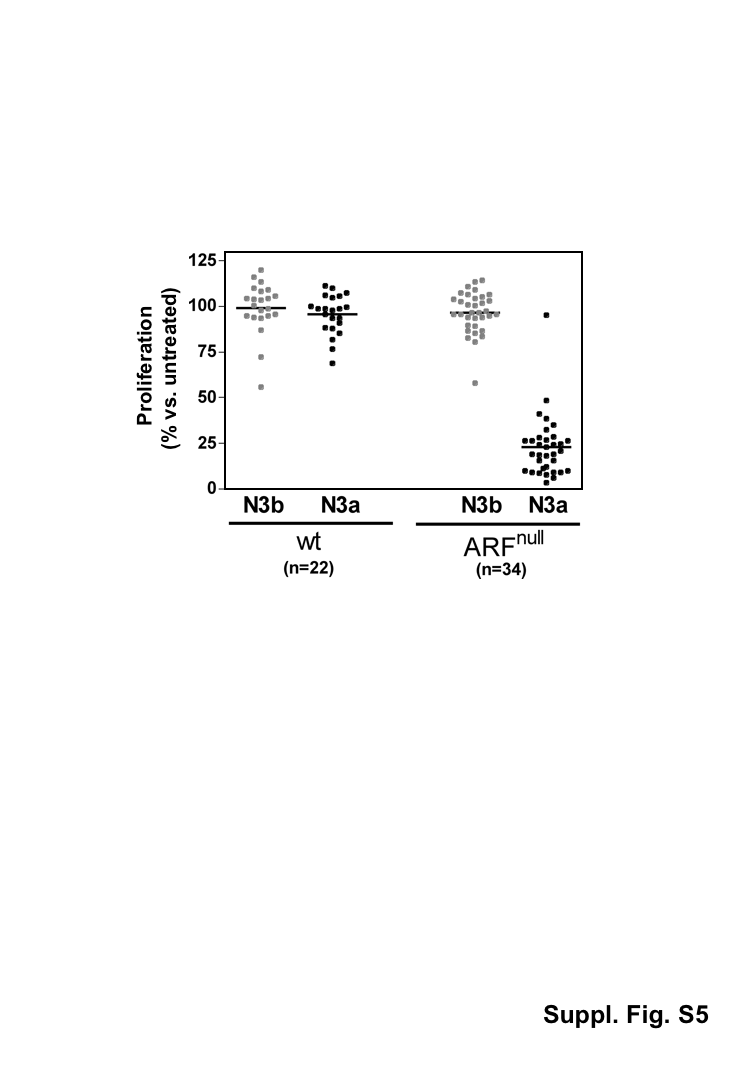

Supplement: Figure S5 — Arf-deficiency relieves the selective pressure to inactivate p53 in 3MC-fibrosarcomas (nutlin-sensitivity assay). Fibrosarcoma cell lines obtained from the wt or Arf-null mice were treated with 10 µM of the active enantiomer of nutlin (nutlin-3a, N3a) or with the inactive enantiomer (nutlin-3b, N3b). Nutlin activates p53 by inhibiting MDM2. After 48 h, cells were fixed and the proportion of S-phase cells was determined by flow cytometry. All the fibrosarcoma cell lines derived from wt mice did not respond to N3b, thus indicating that p53 was not functional; in contrast, all but one cell lines derived from Arf-null mice responded to N3b by strongly decreasing proliferation. (0.07 MB TIF) [file pone.0005475.s005.tif]

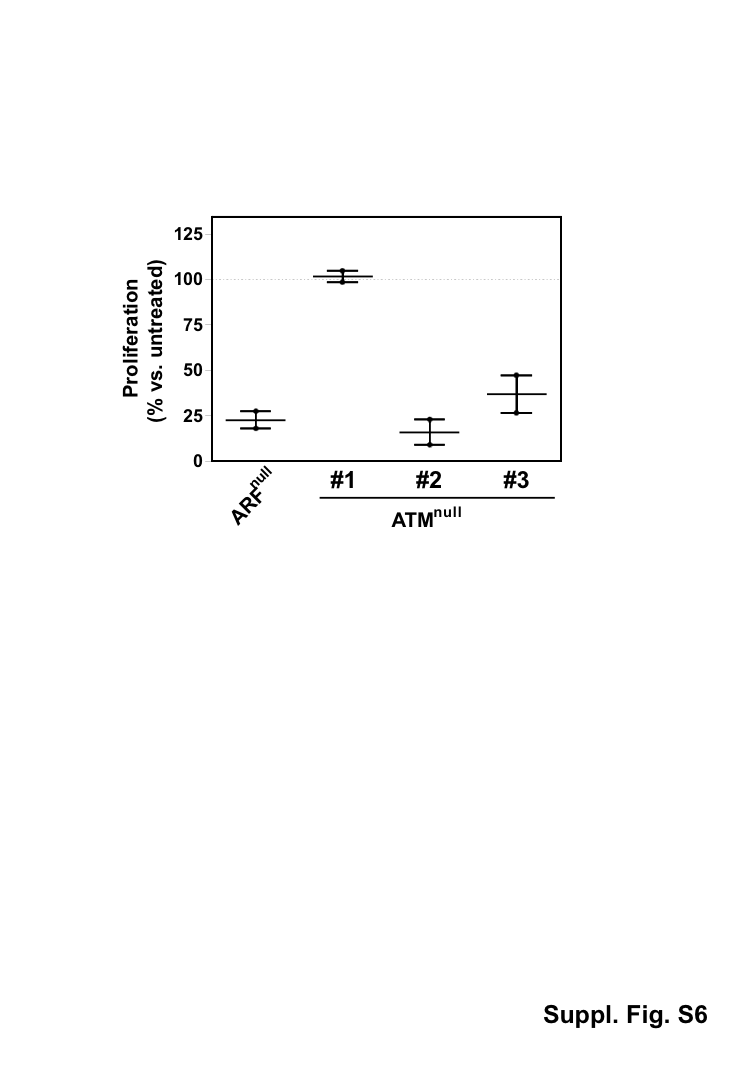

Supplement: Figure S6 — Identification of Atm-null fibrosarcoma cell lines retaining functional p53 (nutlin-sensitivity assay). The large majority of 3MC-fibrosarcomas from Atm-null mice lacked functional p53 (see Table 1), however, two Atm-null cell lines were identified that retained a functional p53. These cell lines, #2 and #3 in the figure, responded to 10 µM nutlin (racemic mixture) by undergoing cell cycle arrest as measured by flow cytometry 48 h after treatment. (0.06 MB TIF) [file pone.0005475.s006.tif]
