# Supplementary figures and images for: Correction: Limited Role of Murine ATM in Oncogene-Induced Senescence and p53-Dependent Tumor Suppression
Source: PLoS One. 2024 Feb 1;19(2):e0298441. doi: 10.1371/journal.pone.0298441 (PMC10833544; doi:10.1371/journal.pone.0298441)

## Slide 1
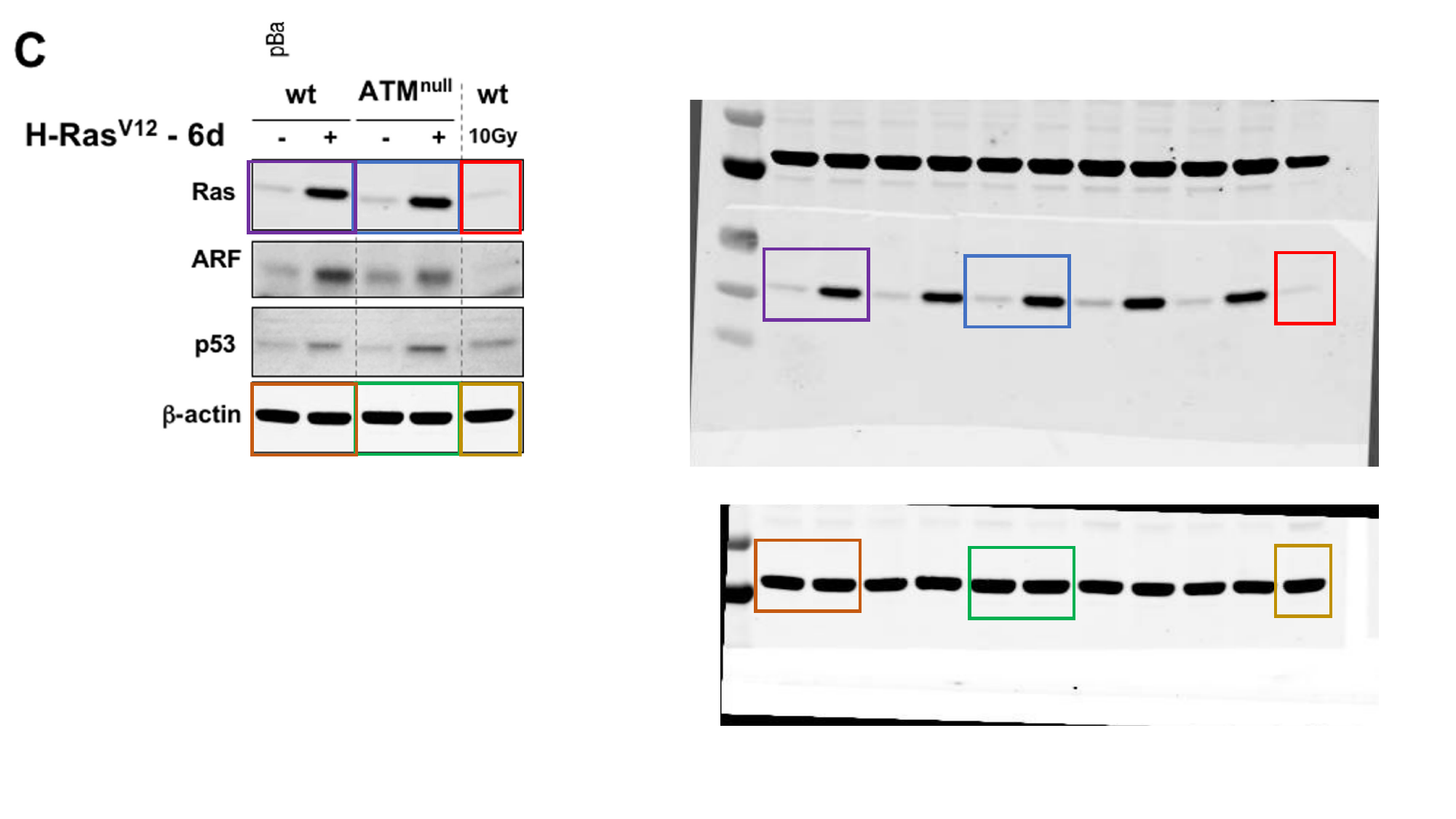

## Slide 2
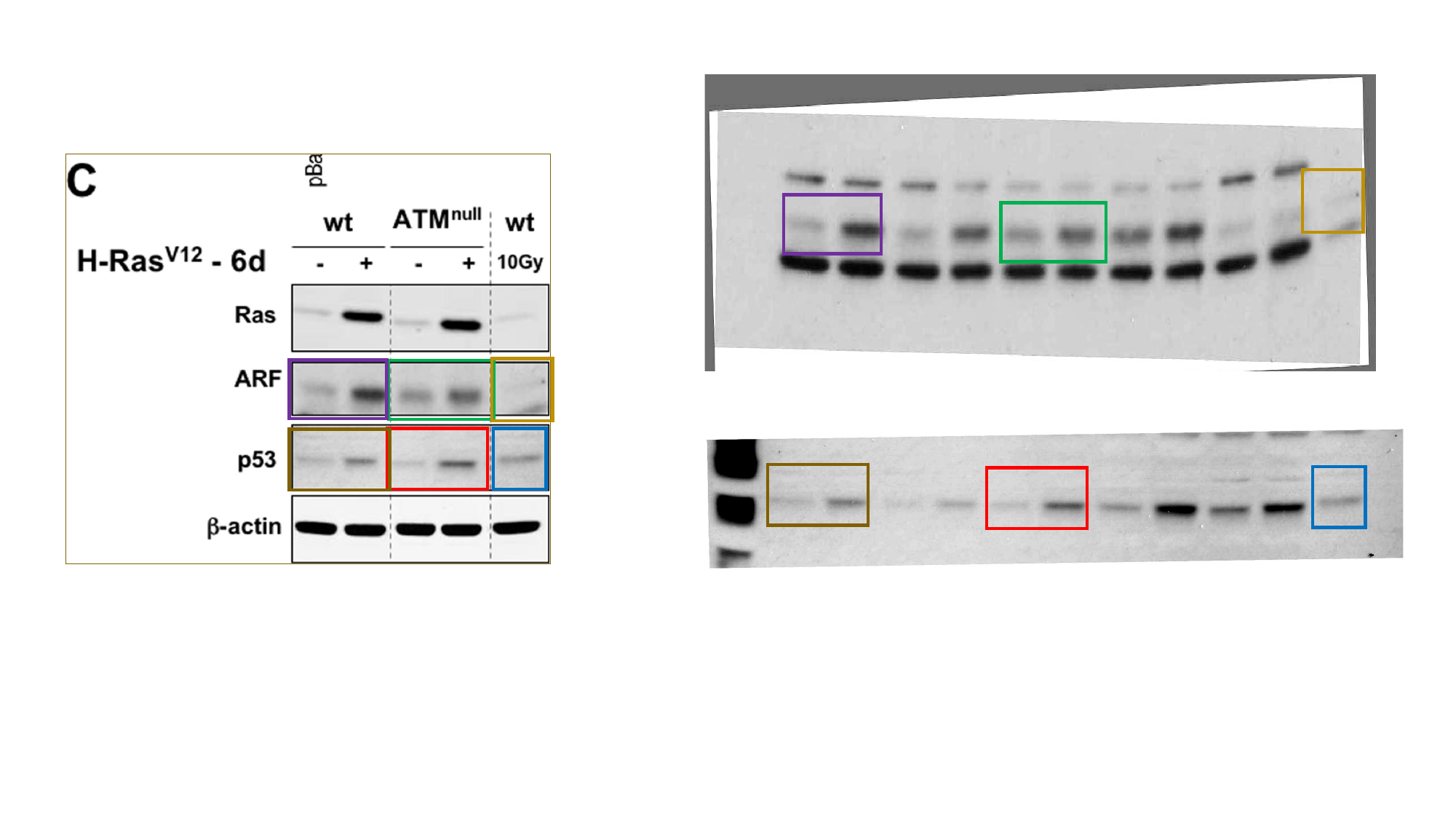

Supplement: S1 File — (PPTX) [file pone.0298441.s001.pptx]

## Slide 1
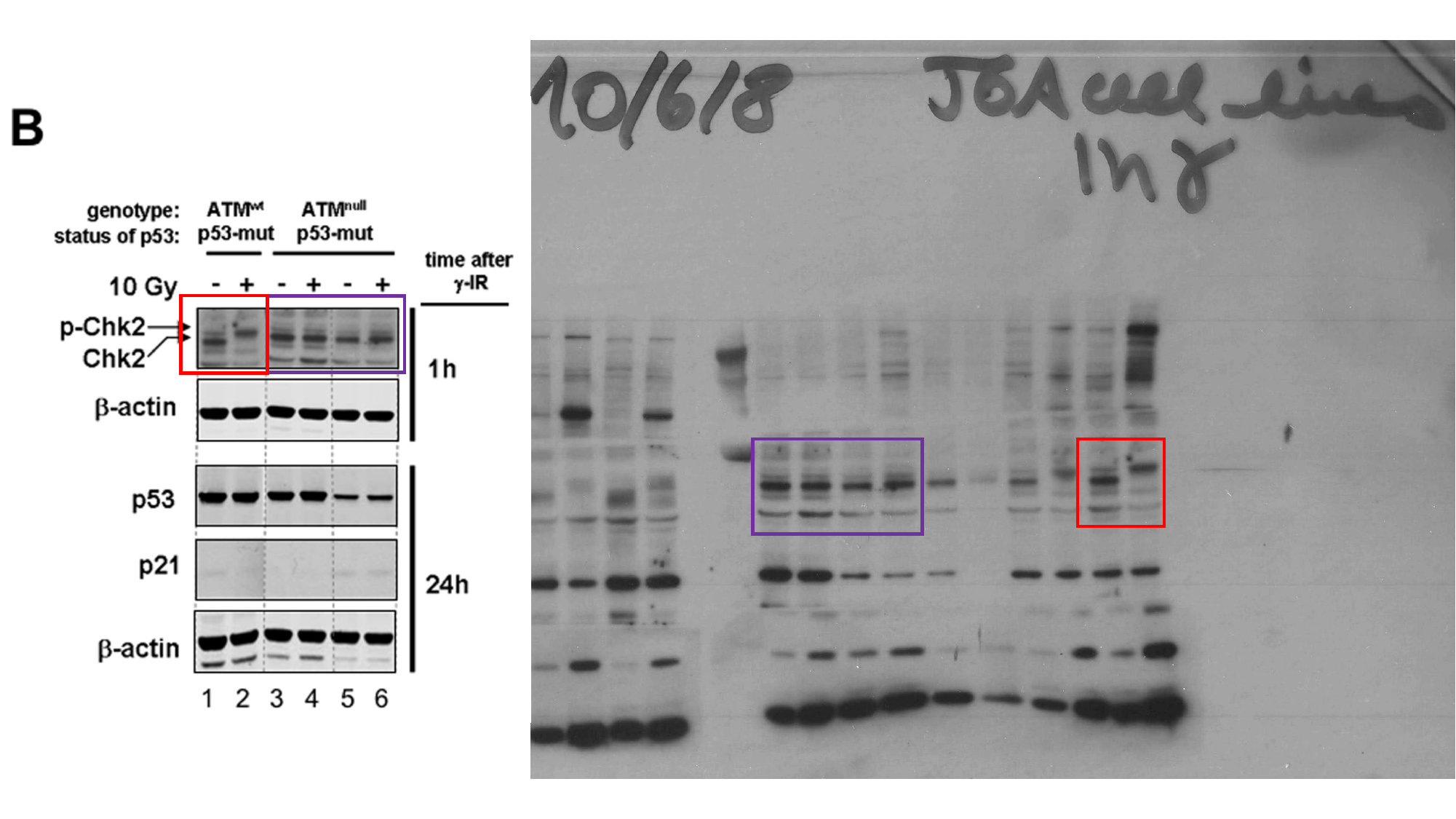

## Slide 2
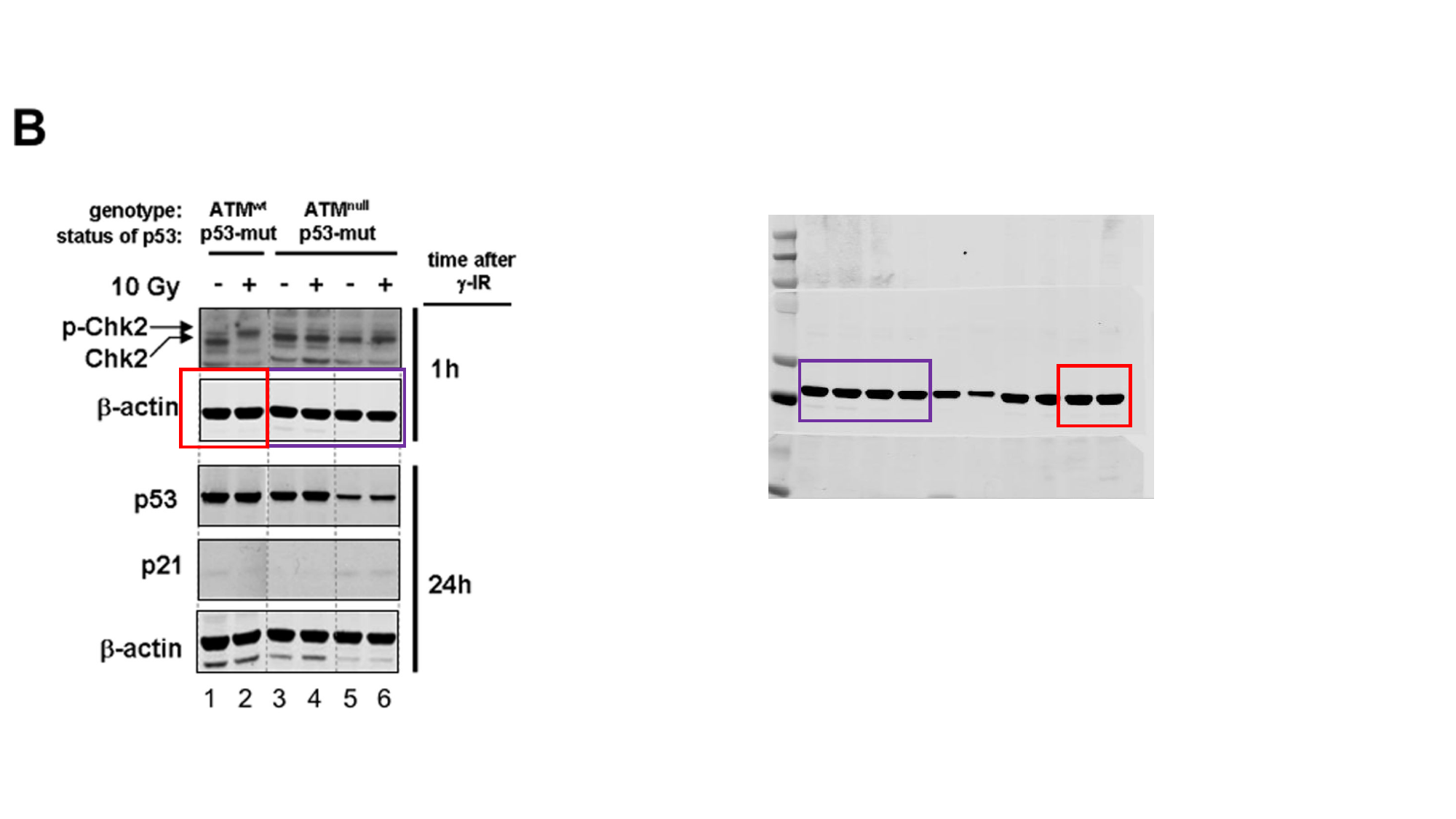

## Slide 3
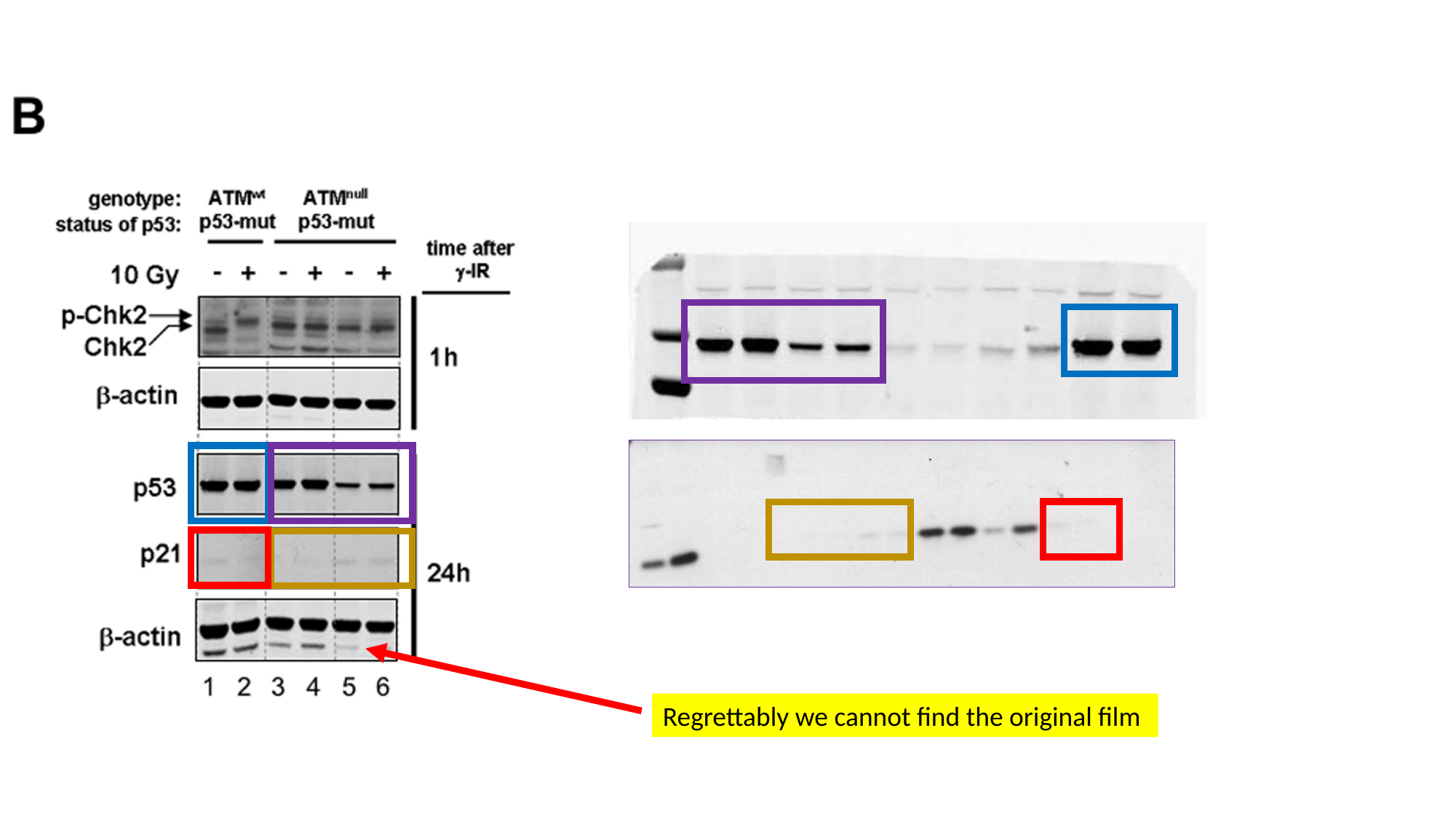

Regrettably we cannot find the original film

## Slide 4
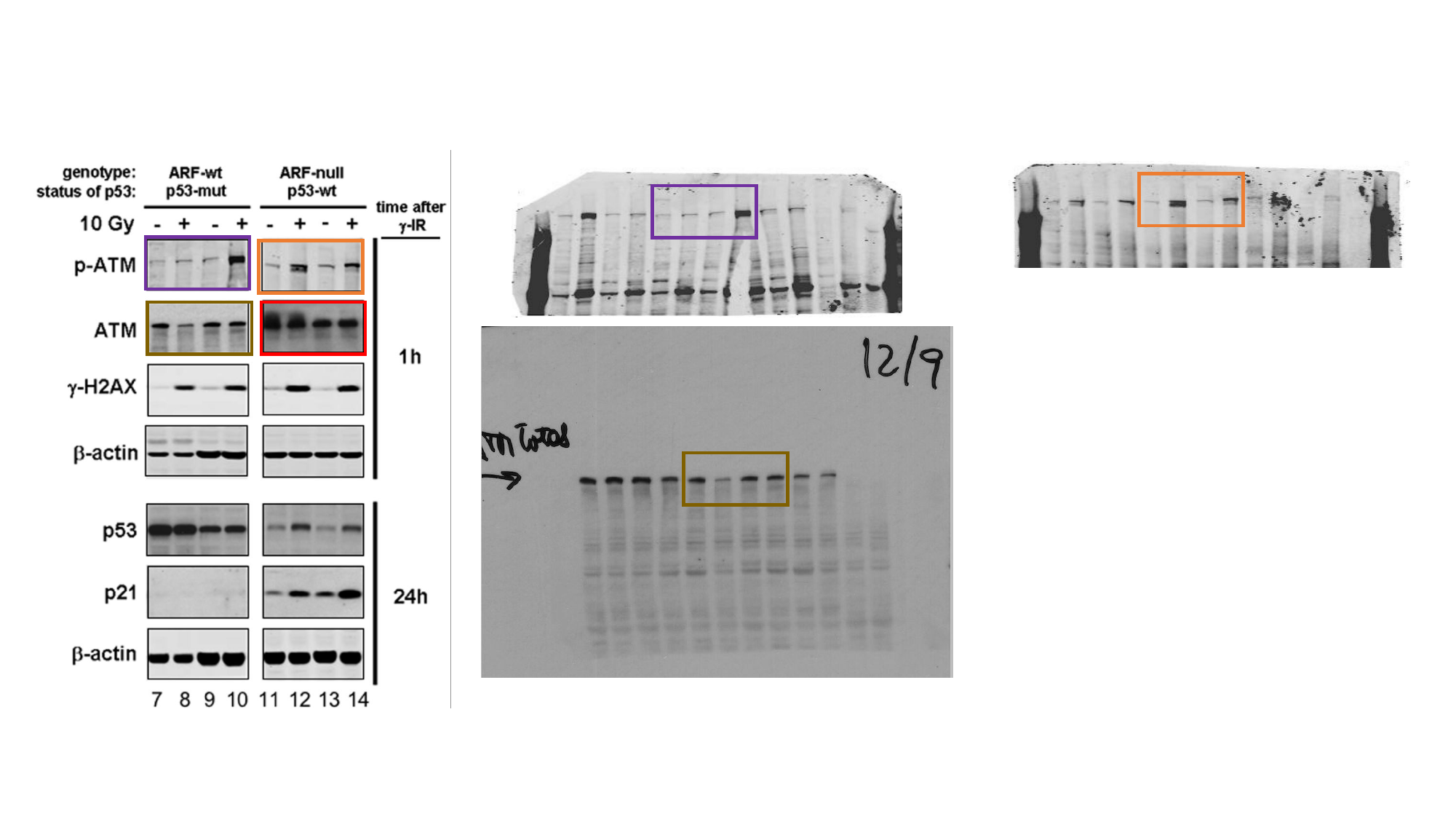

## Slide 5
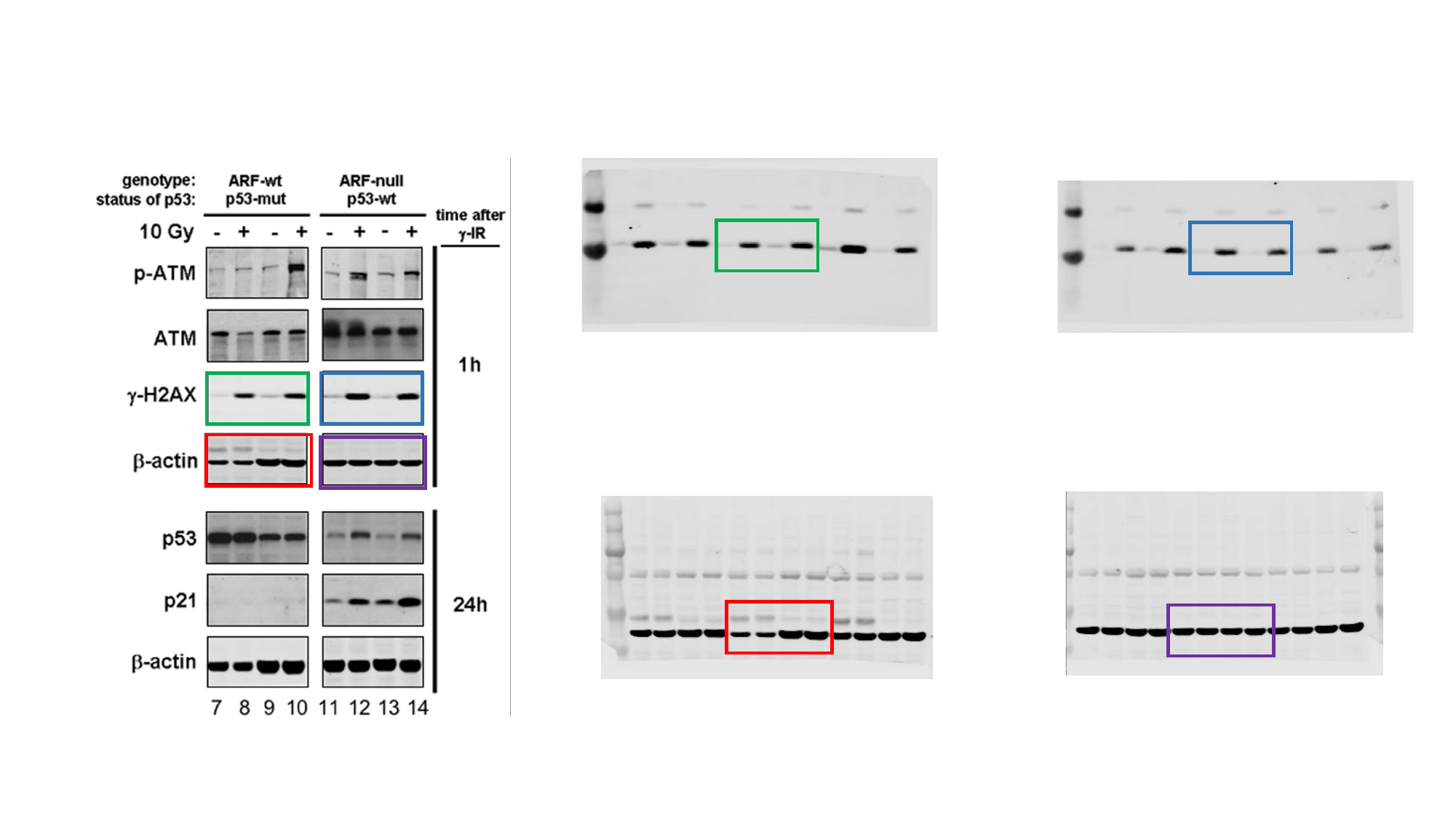

## Slide 6
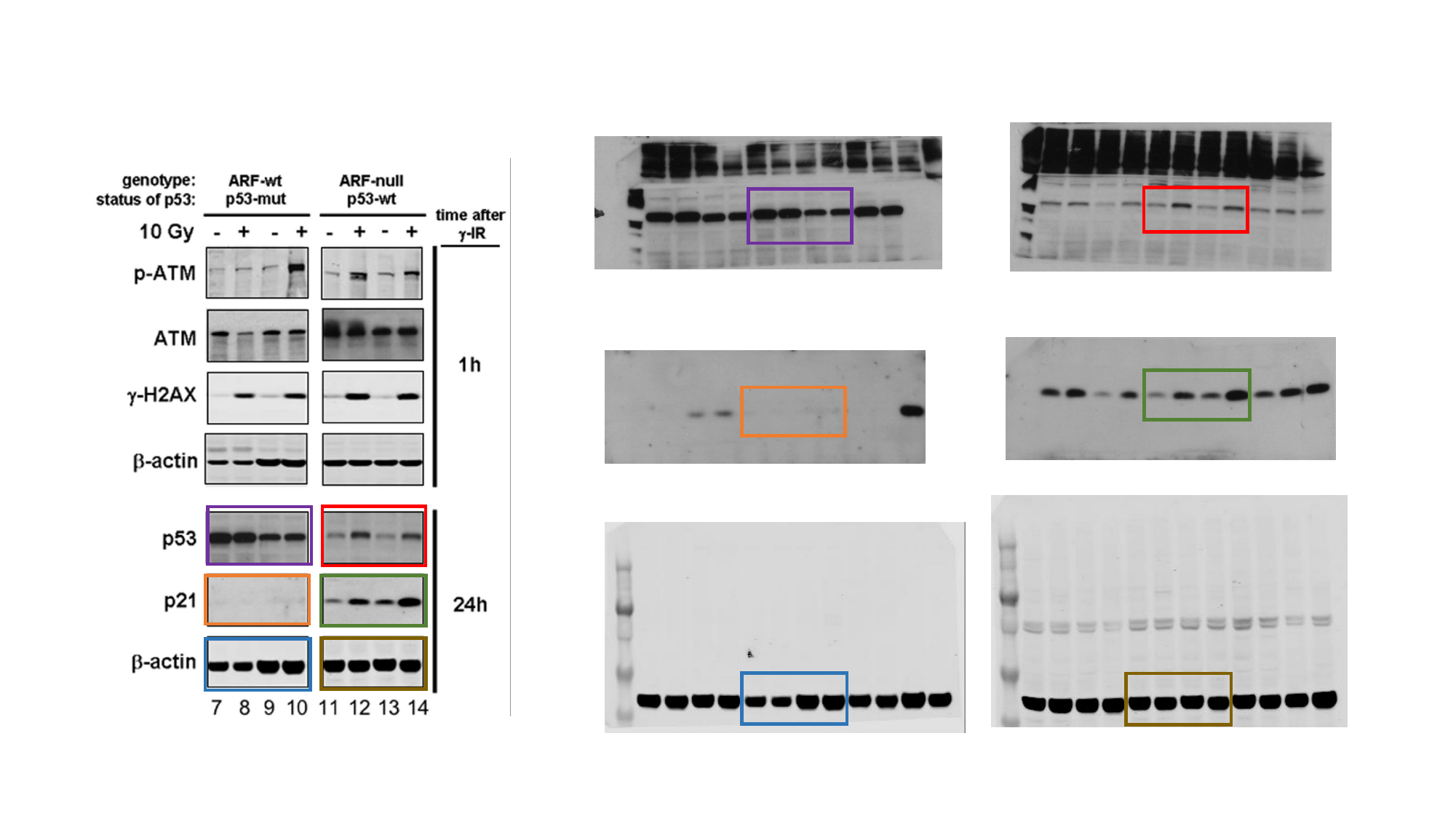

Supplement: S2 File — (PPTX) [file pone.0298441.s002.pptx]
